# The value of deep learning-based X-ray techniques in detecting and classifying K-L grades of knee osteoarthritis: a systematic review and meta-analysis

**DOI:** 10.1007/s00330-024-10928-9

**Published:** 2024-07-12

**Authors:** Haoming Zhao, Liang Ou, Ziming Zhang, Le Zhang, Ke Liu, Jianjun Kuang

**Affiliations:** 1 Hunan University of Chinese Medicine, 300 Xueshi Road Hanpu Science & Education Park, Yuelu District, Changsha, Hunan 410208 China; 2 Hunan Academy of Chinese Medicine No. 142 Yuehua Road, Yuelu District, Changsha, Hunan 410013 China

**Keywords:** Knee osteoarthritis, X-ray, Deep learning, Kellgren-Lawrence grading system, Meta-analysis

## Abstract

**Objectives:**

Knee osteoarthritis (KOA), a prevalent degenerative joint disease, is primarily diagnosed through X-ray imaging. The Kellgren-Lawrence grading system (K-L) is the gold standard for evaluating KOA severity through X-ray analysis. However, this method is highly subjective and non-quantifiable, limiting its effectiveness in detecting subtle joint changes on X-rays. Recent researchers have been directed towards developing deep-learning (DL) techniques for a more accurate diagnosis of KOA using X-ray images. Despite advancements in these intelligent methods, the debate over their diagnostic sensitivity continues. Hence, we conducted the current meta-analysis.

**Methods:**

A comprehensive search was conducted in PubMed, Cochrane, Embase, Web of Science, and IEEE up to July 11, 2023. The QUADAS-2 tool was employed to assess the risk of bias in the included studies. Given the multi-classification nature of DL tasks, the sensitivity of DL across different K-L grades was meta-analyzed.

**Results:**

A total of 19 studies were included, encompassing 62,158 images. These images consisted of 22,388 for K-L_0_, 13,415 for K-L_1_, 15,597 for K-L_2_, 7768 for K-L_3_, and 2990 for K-L_4_. The meta-analysis demonstrated that the sensitivity of DL was 86.74% for K-L_0_ (95% CI: 80.01%–92.28%), 64.00% for K-L_1_ (95% CI: 51.81%–75.35%), 75.03% for K-L_2_ (95% CI: 66.00%–83.09%), 84.76% for K-L_3_ (95% CI: 78.34%–90.25%), and 90.32% for K-L_4_ (95% CI: 85.39%–94.40%).

**Conclusions:**

The DL multi-classification methods based on X-ray imaging generally demonstrate a favorable sensitivity rate (over 50%) in distinguishing between K-L_0_-K-L_4_. Specifically, for K-L_4_, the sensitivity is highly satisfactory at 90.32%. In contrast, the sensitivity rates for K-L_1-2_ still need improvement.

**Clinical relevance statement:**

Deep-learning methods have been useful to some extent in assessing the effectiveness of X-rays for osteoarthritis of the knee. However, this requires further research and reliable data to provide specific recommendations for clinical practice.

**Key Points:**

- *X-ray deep-learning (DL) methods are debatable for evaluating knee osteoarthritis (KOA) under The Kellgren-Lawrence system (K-L).*
- *Multi-classification deep-learning methods are more clinically relevant for assessing K-L grading than dichotomous results.*
- *For K-L3 and K-L4, X-ray-based DL has high diagnostic performance; early KOA needs to be further improved.*

## Introduction

Knee osteoarthritis (KOA) is a common orthopedic disease, primarily causing pain, stiffness, reduced joint mobility, and gait disturbances. These symptoms not only exacerbate the disease but also lead to physical limitations and disability in older people [1, 2]. Clinically, KOA is characterized by the gradual deterioration of articular cartilage at the ends of bones and structural changes in joint tissues, including deformed bones and cartilage [3]. Radiological signs include osteophyte formation, joint space narrowing (JSN), and subchondral sclerosis. Currently, there are no curative treatments for KOA. Although pharmaceutical options like non-steroidal anti-inflammatory drugs and central analgesics can relieve pain and improve functions, their significant side effects, especially with prolonged use, are noteworthy. Jonathon Charlesworth et al highlight the potential risks and aggravated conditions associated with extended use of these medications [4]. Consequently, early diagnosis of KOA is crucial.

X-ray imaging is a prominent diagnostic tool for KOA, valued for its safety, efficiency, cost-effectiveness, and widespread use [5]. The Kellgren-Lawrence grading system (K-L), endorsed by the World Health Organization since 1961 as the gold standard for assessing KOA severity [6], categorizes this condition into five levels (0 to 4), with higher numbers indicating more severe damage (Table 1).

**Table 1:** K-L grading system

K-L grades	OA analysis
Grade 0	Normal knee
Grade 1	Suspected OA (suspicious joint space narrowing with possible osteophyte formation)
Grade 2	Mild OA (possible joint space narrowing, definite osteophyte formation)
Grade 3	Moderate OA (multiple osteophyte formations, definite joint space narrowing, subchondral sclerosis)
Grade 4	Severe OA (marked joint space narrowing, osteophytes, definite bone deformity, severe sclerosis)

However, grading is currently largely contingent on the experience and judgment of clinicians. This grading method is subjective and may be influenced by other confounding factors, possibly resulting in misclassification and thus affecting clinical decisions and the recovery of patients.

With the continual advancement of computer technology and the refinement of statistical theories, deep learning (DL) has been increasingly adopted in clinical practices. DL, which learns features directly from data, transforms medical image analysis. In this context, some studies have constructed radiomics-based DL for the diagnosis and grading of KOA [7, 8]. Nonetheless, these DL models yield inconsistent results, and the sensitivity and diagnostic value of artificial intelligence remain elusive, undoubtedly impeding the research on artificial intelligence in this domain. Consequently, this study aims to assess the efficiency of X-ray-based artificial intelligence in the non-invasive detection and classification of KOA.

## Methods

### Study registration

This research was conducted in accordance with the guidelines for systematic reviews and meta-analyses and was prospectively registered on PROSPERO (ID: CRD42023450313).

### Eligibility criteria

Inclusion criteria

1. Studies involving patients with KOA.
2. Studies that have fully constructed DL for identifying K-L grading of KOA based on X-ray images.
3. Studies using different DL approaches but published from the same dataset are included in this systematic review.
4. The types of studies included in this systematic review are case-control studies, cross-sectional studies, and cohort studies.
5. Studies reported in the English language.

Exclusion criteria

1. Studies with significant problems regarding the diagnosis and severity definition of KOA.
2. Studies that construct DL models are not based on X-ray images.
3. Studies where the model employed is not DL.
4. Studies with a sample size of fewer than 20 cases, or those lacking an independent validation or test set.
5. Conference abstracts that have been publicly released without peer review.

### Data sources and search strategy

A systematic search was conducted across databases including PubMed, Cochrane, Embase, Web of Science, and IEEE. The search formulas were composed of topic terms and text words. No restrictions were imposed on region or publication year. The search was completed up to July 11, 2023. Detailed search strategies are provided in Table S1.

### Study selection and data extraction

The retrieved literature was imported into EndNote. Following the removal of duplicates, titles, and abstracts were screened to identify preliminarily eligible studies. Subsequently, their full texts were downloaded. By comprehensively reading these full texts, studies that ultimately met the requirements of this systematic review were identified. Prior to extracting the data, a standardized electronic template was prepared. The extracted items including title, first author, publication year, country of the authors, study type, patient source, image source, severity measurement standards, the number of cases for each severity level, total case count, total cases in the training set, method of generation for validation and test sets, external validation, number of cases in the validation set, number of cases in the test set, type of model used, and whether comparisons were made with clinical doctors. The aforementioned literature screening and data extraction were independently performed by two researchers, Le Zhang and Ziming Zhang, and were cross-checked upon completion. In cases of disagreement, a third researcher, Liang Ou, was consulted.

### Assessment of study quality

QUADAS-2 [9, 10], as a modified version of the Quality Assessment of Diagnostic Accuracy Studies (QUADAS) tool, consists of four components: patient selection, index test, reference standard, and flow and timing. Each component encompasses several specific questions. The answer for each question is “yes”, “no”, or “unclear”, indicating a “low”, “high”, or “unclear” risk of bias, respectively. If all key questions within a domain receive a “yes” answer, the risk of bias can be assessed as low.

However, if even one question within an informative domain is answered with a “no”, the potential for bias exists, necessitating the evaluators to assess the risk of bias based on pre-established criteria. If the included studies lack sufficient details for evaluators to make a definitive judgment, the risk of bias is considered unclear.

### Synthesis methods

Data from independent validation and test sets were meta-analyzed, respectively. Given that there are five levels in the K-L grading for KOA, we summarized the sensitivity of DL for each individual grade.

For the meta-analysis of sensitivity, data would be transformed based on the following scenarios:

i. No transformation is required if the sensitivity rate of all samples is between 20% and 80%.
ii. The logit transformation is applied when rates are less than 20% or greater than 80%.
iii. In cases where a significant number of values are at 0% or/and 100%, the double-arcsine transformation is used [11, 12].

The model for meta-analysis is selected based on the heterogeneity index (*I*^*2*^). A random-effects model is adopted when *I*^*2*^ exceeds 50%, whereas a fixed-effects model is used when *I*^*2*^ is less than 50%. Funnel plots offer a visual representation of publication bias across various indicators within studies. The Egger’s test is employed to statistically examine publication bias. A difference is considered statistically significant if *p* < 0.05. Forest plots for each level and the forest plots for misdiagnosis rates at different levels were generated (Fig. S2–Fig. S21). All meta-analysis results relate to the “metafor” and “meta” packages of R (version 4.2.3).

## Results

### Study selection

**Fig. 1:**
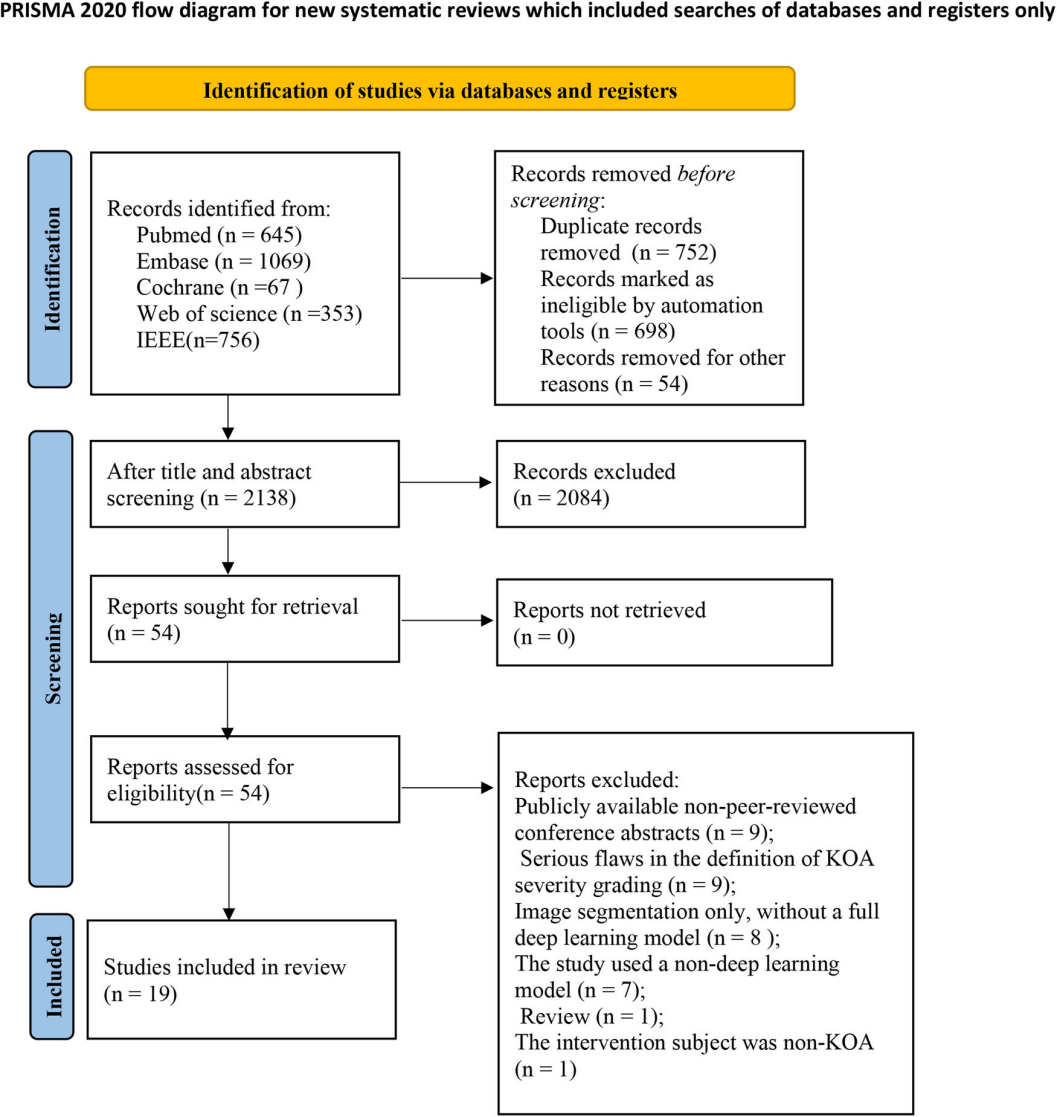
Flowchart of the literature screening process

A total of 2890 articles were retrieved from the database. Among these, 752 were identified as duplicate studies, with 698 automatically marked as duplicates by the reference management software. After reviewing the titles and abstracts, 2138 articles remained. Based on the eligibility criteria, 2084 ineligible articles were further excluded. Fifty-four articles were initially shortlisted for full-text reading. Upon detailed review, the following 35 studies were excluded: **①** Publicly published, non-peer-reviewed conference abstracts (*n* = 9); **②** Studies with seriously flawed definitions of KOA severity grading (*n* = 9); **③** Articles that focused solely on image segmentation without developing a comprehensive DL model (*n* = 8); **④** Research employing models other than DL (*n* = 7); **⑤** Reviews (*n* = 1); **⑥** Interventions not targeting KOA (*n* = 1). Ultimately, 19 articles [2, 7, 13–29] were eligible and included (shown in Fig. 1).

### Study characteristics

All 19 included articles focused on multi-class DL, comprising a total of 62,158 images. Specifically, there are 22,388 images for K-L_0_, 13,415 for K-L_1_, 15,597 for K-L_2_, 1168 for K-L_3_, and 2990 for K-L_4_. Four studies were published in China [7, 13, 28, 29] and the USA [2, 14–16], respectively. Three articles were published in Pakistan [17–19], while two were published in Saudi Arabia [20, 21], India [22, 23], and South Korea [24, 25], respectively. Meanwhile, one study was published in Morocco [26] and Iraq [27], respectively. Among them,15 are case-control studies [2, 14–24, 27–29] three are retrospective cohort studies [7, 13, 25], and one is a prospective study [26] Six studies [7, 13–16, 22]compared DL with clinical physicians. Data for eight studies came from OAI [2, 14, 15, 18, 20, 23, 27, 28], one from MOST [16], one from both OAI and MOST [26], six from the hospital’s PACS system [7, 13, 17, 22, 25, 29], one from a prior study [24], and two did not specify their data source [19, 21]. The detailed characteristics of the included studies are shown in Table 2.

**Table 2:** Basic characteristics of the included studies

No.	First author and year of publication	Country of authors	Study type	Patient source	Age	Gender	Number of cases by severity	Total sample size	Number of cases in the training set	Generation methods of validation set and test set	Number of cases in the validation set	Number of cases in the test set	Model type	Whether or not to compare with clinicians	Comparison between deep learning and clinicians
1	Yassine Nasser (2023) [26]	Morocco	Prospective	MOST and OAI	Age range: aged 50 to 79 years	Male: 3015 (MOST) OAI: 4796 (gender-neutral)	MOST:0:6008 (I) 1:2933 (I) 2:3045 (I) OAI:0:1116 (I) 1:513 (I) 2:806 (I)	MOST:3015 (P) OAI:4796 (P)	MOST:0:6008 (I) 1:2933 2:3045	Randomization	OAI:0:1116 (I) 1:513 2:806	OAI:0:2313 (I) 1:1071 (I) 2:1545 (I)	DST-CNN	No	None
2	Nan Chen (2022) [28]	China	Case-control	OAI and PACS	Age range: 45 to 79 years	None	0:2471OAI/120XY (I) 1:1071/138 (I) 2:1230/117 (I) 3:871/96 (I) 4:204/29 (I)	OAI:5847 (I) Depart of Xiang ya:500 (I)	None	None	None	None	YOLOv3	No	None
3	Rabbia Mahum (2021) [17]	Pakistan	Case-control	MultiCentre	None	None	0:100 (I)	None	None	A five-fold validation was used, such as (50, 50), (25, 75), (30, 70), (40, 60), (20, 80)	None	None	CNN	No	None
4	Berk Norman (2018) [14]	USA	Case-control	OAI	Age = 61.2 ± 9.2 years	Male:Female = 1886:2618	0:16044 (I) 1:7514 (I) 2:9421 (I) 3:5124 (I) 4:1490 (I)	OAI:39,593 (I) Subjects:4500 (I)	25,873 (I)	Randomization (65:20:15)	7779 (I)	5941 (I)	DenseNet neural network	Yes	Radiologist consent for AI classifications: No OA: 9.1% Mild OA: 75% Moderate OA: 66.7%
5	Kevin A. Thomas (2019) [15]	USA	Case-control	OAI	Age range: 45–79 years Average age: Female: 60.9 years Male: 61.3 years	Male:female = 42%: 58% (2615:1893)	None	4508 (P) 40,280 (I)	3606 (P) 32,116 (I)	Random sampling	450 (P) 4074 (I)	452 (P) 4090 (I)	A 169-layer convolutional neural network	Yes	Accuracy: Radiologist: 0.840 (42/50) The model: 0.90 (45/50)
6	Jianfeng Yang (2022) [7]	China	Retrospective	Single Center (a hospital in Shanghai, China)	Age range: age ≥ 40 years	Gender-neutral	0:53 (I) 1:283 (I) 2:409 (I) 3:1496 (I) 4:1462 (I)	2579 (I) 2378 (P)	1598 (I) 1532 (P)	Randomization	158 (I) 149 (P)	823 (I) 697 (P)	RefineDet	Yes	The quadratic weighted Kappa coefficient: 0.815 (*p* < 0.01, 0.727–0.903); The average quadratic weighted Kappa coefficient : 0.853 (*p* < 0.01, 95% CI 0.769–0.936).
7	Ganesh Kumar M (2023) [23]	India	Case-control	OAI	None	None	0: 639 (I) 1:296 (I) 2:447 (I) 3:223 (I) 4:51 (I)	4130 (I) 8260 (knee joint)	413 (I) 826 (knee joint)	Random sampling (validation set: training set: test set 7:1:2)	5782 (knee joint) 2891 (I)	828 (I) 1656 (knee joint)	CNN Inception Net V2	No	None
8	S. Sheik Abdullah [22]	India	Case-control	Radiological Center (KGS scan center, Madurai)	Age range: age ≥ 50 years	Males:Females:805 :1207	0: 826ME1/809ME2 (I) 1:895/912 (I) 2:590/583 (I) 3:429/432 (I) 4:432/436 (I)	2012 (P) 3172 (I)	1468 (P) 2221 (I)	Random sampling (training set: validation set: test set 7:1:2)	181 (P) 317 (I)	336 (P) 634 (I)	Faster RCNN + Modified ResNet-50 using transfer learning	Yes	Variations in grade 1 and grade 2.
9	Dong Hyun Kim [25]	Korea	Retrospective cohort study	DICOM and PACS	Age = 62.3 ± 2.8 years	Male:Female = 921:3445	0: 600 (I) 1:755 (I) 2:1113 (I) 3:1319 (I) 4:579 (I)	4366 (I) 3000 (P)	3464 (I)	Stratified sampling (training set: validation set 9:1)	386 (I)	516 (I)	Squeeze-and-excitation ResNet (SE-ResNet) modules	No	None
10	Saleh Hamad Sajaan Almansour [21]	Saudi Arabia	Case-control	None	None	None	0:3253 (I) 1:1495 (I) 2:2175 (I) 3:1086 (I) 4:251 (I)	8381 (I)	5097 (I)	Randomization (training set: validation set: test set 6:1:3)	826 (I)	2458 (I)	CNN InceptionResNet V2	No	None
11	Sang-min Lee (2023) [24]	Korea	Case-control	Previous Studies	None	None	0:3253 (I) 1:1495 (I) 2:2175 (I) 3:1086 (I) 4:251 (I)	8250 (I)	6604 (I)	None	None	1656 (I)	TinyNetA 1 VGG19 2 DenseNet169 MobileVitV2_150	No	None
12	Wei Li (2023) [13]	China	Retrospective cohort study	Single Center (the Fifth Affiliated Hospital of Sun Yat-sen University (Zhuhai, China)	Age range = 18 to 92 years (mean 51.13 ± 15.11 years)	Male:Female = 864:982	0:1994/1595/399 (Ad/TRc/TEc) (I) 1:1063/850/213 (I) 2:630/504/126 (I) 3:360/288/72 (I) 4:153/122/31 (I)	1846 (P) 4200 (I)	3359 (I)	Randomization (training set: test set 8:2)	None	841 (I)	U-Net ResNet-50	Yes	Accuracy: model 4: an radiologist = 0.96:0.86 (McNemar test, *p* < 0.05)
13	Albert Swiecicki [16]	USA	Case-control	Multicentre Osteoarthritis Study (MOST)	None	None	0:7561 (I) 1:2740 (I) 2:3125 (I) 3:3488 (I) 4:1569 (I)	2802 (P) 18,053 (I)	2040 (P) 13,404 (I)	Random sampling	259 (P) 1740 (I)	503 (P) 3359 (I)	R-CNN models	Yes	The average quadratic-weighted Kappa = 0.769
14	Pingjun Chen (2019) [2]	USA	Case-control	OAI	Age range: 45 to 79 years	None	0:639 (knee joint) 1:296 2:447 3:223 4:51	4130 (I) 8260 (knee joint)	2891(I)	Random sampling (training set: validation set: test set 7:1:2)	413 (I)	826 (I)	ResNet VGG DenseNet Inception YOLOv2	No	None
15	Sozan Mohammed Ahmed (2022) [27]	Iraq	Case-control	OAI	Age range: 45 to 79 years	None	0:3857 (I) 1:1770 (I) 2:2578 (I) 3:1286 (I) 4:295 (I)	4796 (P) 9786 (I)	7045 (I)	Random sampling (8:2); the validation set was derived from 10% of the training set.	783 (I)	1958 (I)	Deep Hybrid Learning-I (DHL-I) Deep Hybrid Learning-II (DHL-II).	No	None
16	Tayyaba Tariq (2023) [18]	Pakistan	Case-control	OAI	None	None	0:3857 (I) 1:1770 (I) 2:2578 (I) 3:1286 (I) 4:295 (I)	9786 (I)	6850 (I)	Random sampling (training set: validation set: test set 7:1:2)	978 (I)	1957 (I)	DenseNet-121 DenseNet-161 ResNet-34 VGG-19 Ensemble	No	None
17	Usman Yunus [19]	Pakistan	Case-control	None	None	None	None	OAI: 1888 (P) MOST 683 (P)	None	None	None	None	CNN Alex-net and Darknet-53 models YOLO-v2 ONNX model	No	None
18	Abdul Sami Mohammed [20]	Saudi Arabia	Case-control	OAI and knee osteoarthritis severity grading dataset	None	None	0:3857 (I) 1:1770 (I) 2:2578 (I) 3:1286 (I) 4:295 (I)	9786 (I)	6851 (I)	Random sampling (training set: validation set: test set 7:1:2)	979 (I)	1958 (I)	VGG16 VGG19 ResNet101 MobileNetV2 InceptionResNetV2 DenseNet121	No	None
19	Bin Liu [29]	China	Case-control	Single Center (a hospital in Shanghai)	None	None	0:756/762/770/758/762 (Groups 1–5, I) 1:174/180/192/188/178 (I) 2:880/894/880/860/878 (I) 3:212/214/196/214/212 (I) 4:194/166/178/196/186 (I)	2770 (I)	0:756/762/770/758/762 (groups 1-5, I) 1:174/180/192/188/178(I) 2:880/894/880/860/878(I) 3:212/214/196/214/212(I) 4:194/166/178/196/186(I)	5-fold cross-validation	None	0:756/762/770/758/762 (groups 1–5, I) 1:196/166/178/196/186 (I) 2:54/48/36/40/50 (I)3:50/48/66/48/50 (I) 4:36/64/52/34/44 (I)	FasterR-CNN	No	None

*I* image, *P* patients, *Ad* adult, TRc training cohort, *TEc* testing cohort, *ME1*Medical Expert-I; ME2 Medical Expert-II, *MOST* Multicenter Osteoarthritis Study, *OAI* osteoarthritis Initiative public dataset, *XY* the Department of Radiology, Third Xiangya Hospital, Central South University

### Assessment of study quality

Given that this research is a meta-analysis based on X-ray DL, some included studies were case-control studies, which, from the perspective of participants, may be prone to significant bias. Fortunately, this classification is automated, thereby limiting potential influences on the results. No threshold was set before the assessment. The internationally recognized K-L grading system, a gold standard for assessing the severity of KOA, was employed during the evaluation. This facilitated the grading of KOA severity. The detailed assessment results are illustrated in Fig. S1.

### Meta-analysis

#### Identify *K-L*_*0*_

**Fig. 2:**
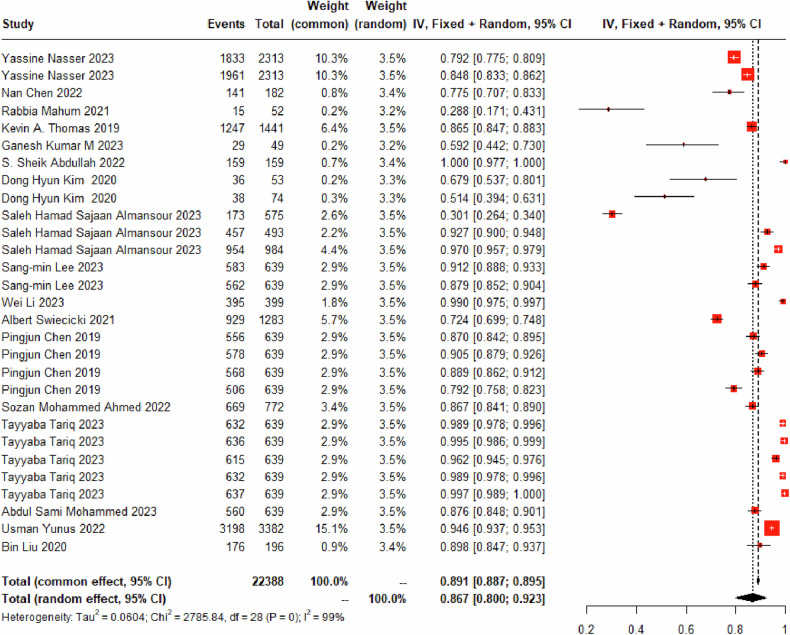
Forest plot for the diagnostic sensitivity of DL based on X-ray for K-L_0_ grade

Twenty-nine DL models for identifying the K-L_0_ grade were included, encompassing 19,745 K-L_0_ grade images. A random-effects model was used, and the pooled diagnostic sensitivity of these models for K-L_0_ was found to be 86.74% (95% CI: 80.01%–92.28%) (Fig. 2 and Table 3). The models misclassified 7.680% (95% CI: 4.32%–11.88%) of images as K-L_1_ grade (Fig. S2), 3.60% (95% CI: 2.12%–5.43%) as K-L_2_ grade (Fig. S3), 0.17% (95% CI: 0%–0.51%) as K-L_3_ grade (Fig. S4), and 0.00% (95% CI: 0.00%–0.00%) as K-L_4_ grade (Fig. S5).

**Table 3:** Confusion matrix of deep learning for K-L grading of KOA (%)

	K-L grade	Predicted KOA levels				
		K-L_0_	K-L_1_	K-L_2_	K-L_3_	K-L_4_
Real KOA levels	K-L_0_ (*n* = 29, sample size = 22,388)	86.74 (80.01–92.28)	7.68 (4.32–11.88)	3.60 (2.12–5.43)	0.17 (0.00–0.51)	0.00 (0.00–0.00)
	K-L_1_ (*n* = 30, sample size = 13,415)	19.80 (12.48–0.2830)	64.00 (51.81–0.7535)	11.24 (7.36–15.80)	1.13 (0.60–1.79)	0.00 (0.00–0.03)
	K-L_2_ (*n* = 30, sample size = 15,597)	6.91 (4.26–10.11)	9.95 (5.86–14.95)	75.03 (66.00–83.09)	4.57 (2.51–7.18)	0.01 (0.00–0.06)
	K-L_3_ (*n* = 29, sample size = 7768)	0.38 (0.01–1.0)	1.55 (0.55–2.96)	7.51 (4.33–11.45)	84.76 (78.34–90.25)	2.76 (1.38–4.52)
	K-L_4_ (*n* = 29, sample size = 2990)	0.00 (0.00–0.01)	0.00 (0.00–0.00)	0.04 (0.00–0.38)	8.05 (4.49–12.39)	90.32 (85.39–94.40)

Note: “*n*” represents the number of validation cohorts, and “sample size” represents the number of images in each KOA level

#### Identify *K-L*_*1*_

**Fig. 3:**
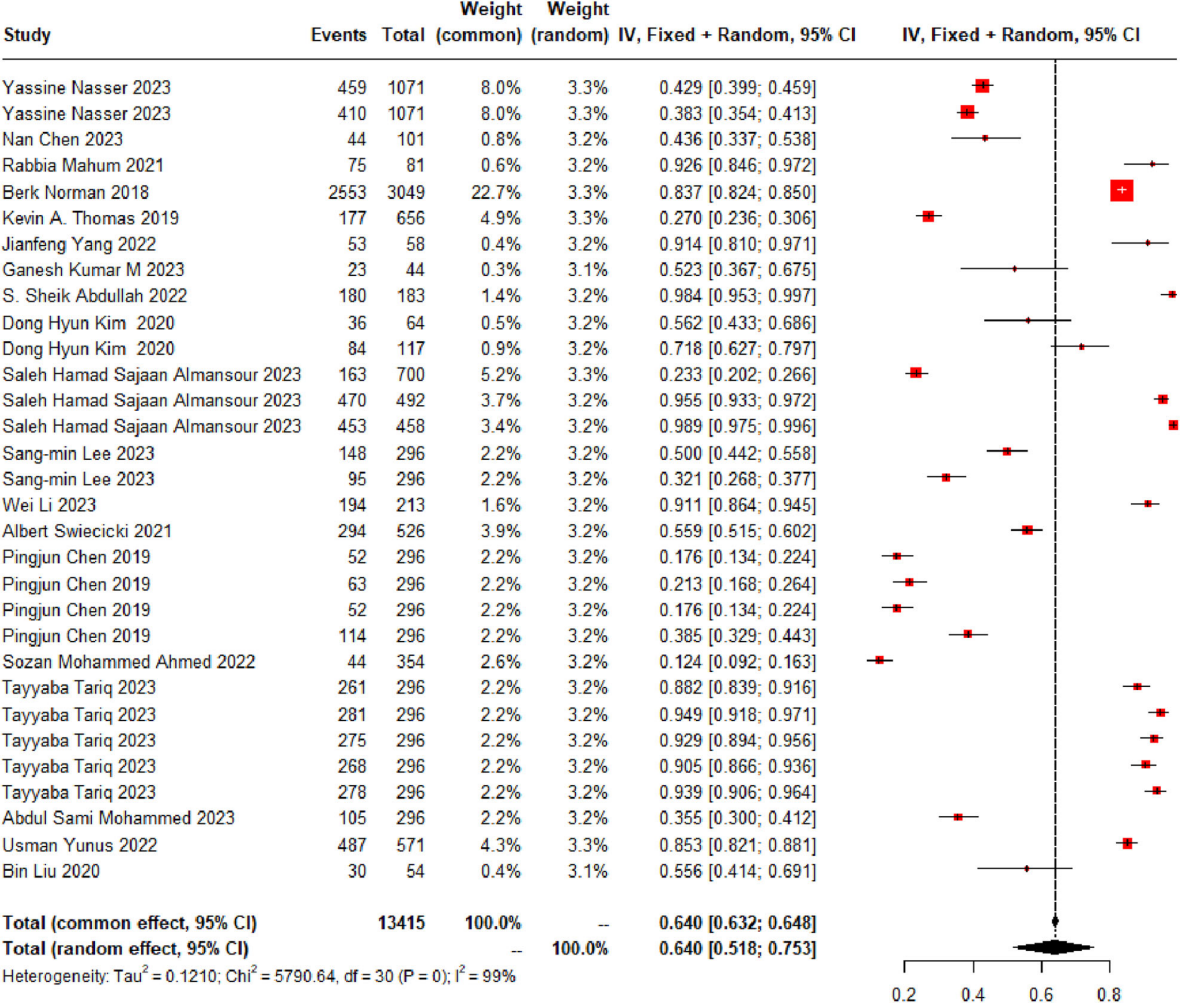
Forest plot of the diagnostic sensitivity for K-L_1_ grade based on DL using X-rays

Thirty DL models for identifying the K-L_1_ grade were included, incorporating 8221 images of the K-L_1_ grade. The random-effects model was utilized, and the models demonstrated a diagnostic sensitivity of 64.00% for the K-L_1_ grade (95% CI: 51.81%–75.35%) (Fig. 3 and Table 3). The misdiagnosis rates were as follows: 19.80% (95% CI: 12.48%–28.30%) were misclassified as K-L_0_ grade (Fig. S6), 11.24% (95% CI: 7.36%–15.80%) as K-L_2_ grade (Fig. S7), 1.13% (95% CI: 0.60%-1.79%) as K-L_3_ grade (Fig. S8), and 0.00% (95% CI: 0.00%–0.03%) as K-L_4_ grade (Fig. S9).

#### Identify *K-L*_*2*_

**Fig. 4:**
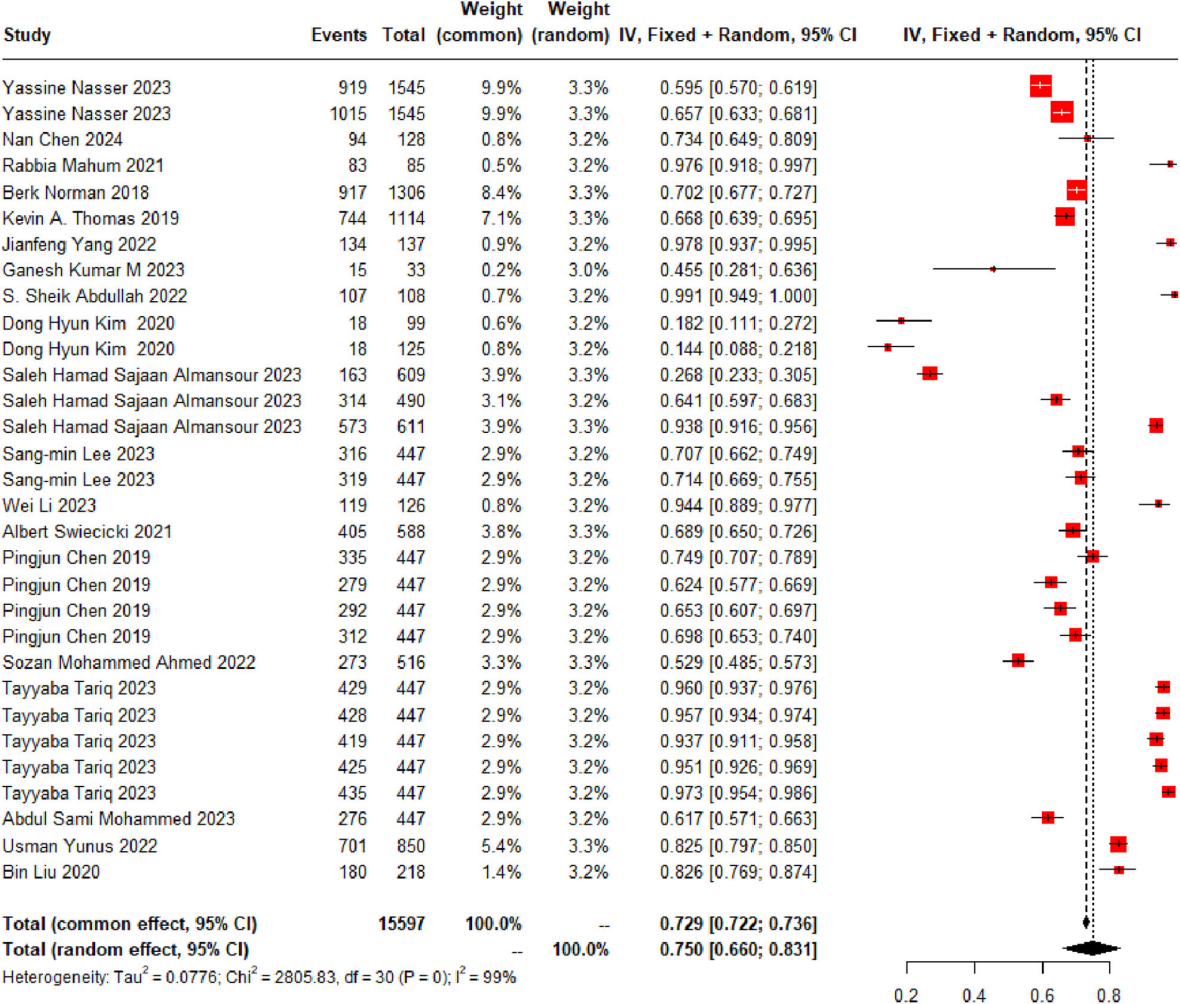
Forest plot illustrating the diagnostic sensitivity of K-L_2_ grade using DL based on X-rays

Thirty DL models for identifying the K-L_2_ grade were included, incorporating 11,057 images of the K-L_2_ grade. By employing a random-effects model, the aggregated diagnostic sensitivity of these models for K-L_2_ was found to be 75.03% (95% CI: 66.00%–83.09%) (Fig. 4 and Table 3). Misclassification rates were identified as follows: 6.91% (95% CI: 4.26%–10.11%) were erroneously categorized as K-L_0_ grade (Fig. S10), 9.95% (95% CI: 5.86%–14.95%) as K-L_1_ grade (Fig. S11), 4.57% (95% CI: 2.51%–7.18%) as K-L_3_ grade (Fig. S12), and 0.01% (95% CI: 0.00%–0.06%) as K-L_4_ grade (Fig. S13).

#### Identify *K-L*_*3*_

**Fig. 5:**
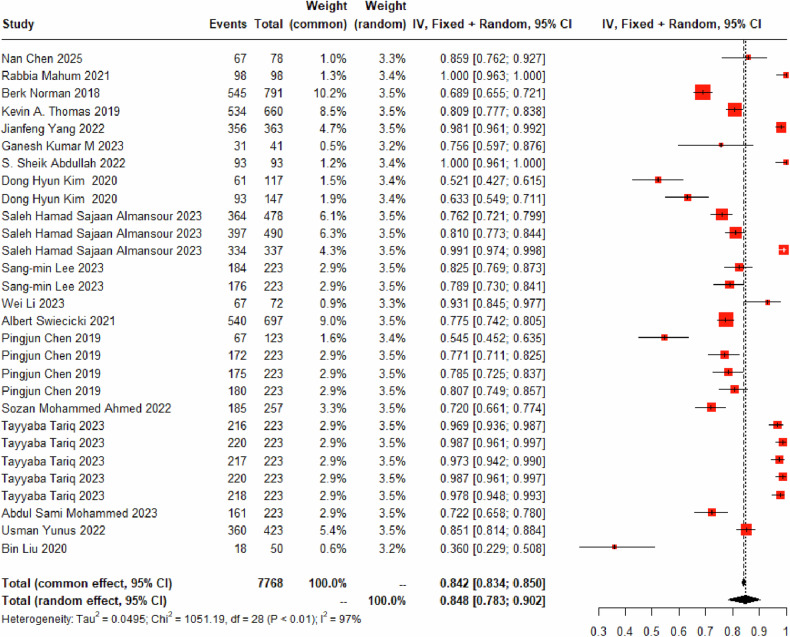
Forest plot representing the diagnostic sensitivity of K-L_3_ grade via DL based on X-ray imagery

Twenty-nine DL models were utilized to identify the K-L_3_ grade, incorporating 6,349 K-L_3_ images. A random-effects model was used for data synthesis, and the pooled diagnostic sensitivity of these models for the K-L_3_ grade was determined to be 84.76% (95% CI: 78.34%–90.25%) (Fig. 5 and Table 3). The misclassification rates for this grade were as follows: 0.38% (95% CI: 0.01%–1.04%) for K-L_0_ grade (Fig. S14), 1.55% (95% CI: 0.55%–2.96%) for K-L_1_ grade (Fig. S15), 7.51% (95% CI: 4.33%–11.45%) for K-L_2_ grade (Fig. S16), and 2.76% (95% CI: 1.38%–4.52%) for K-L_4_ grade (Fig. S17).

#### Identify *K-L*_*4*_

**Fig. 6:**
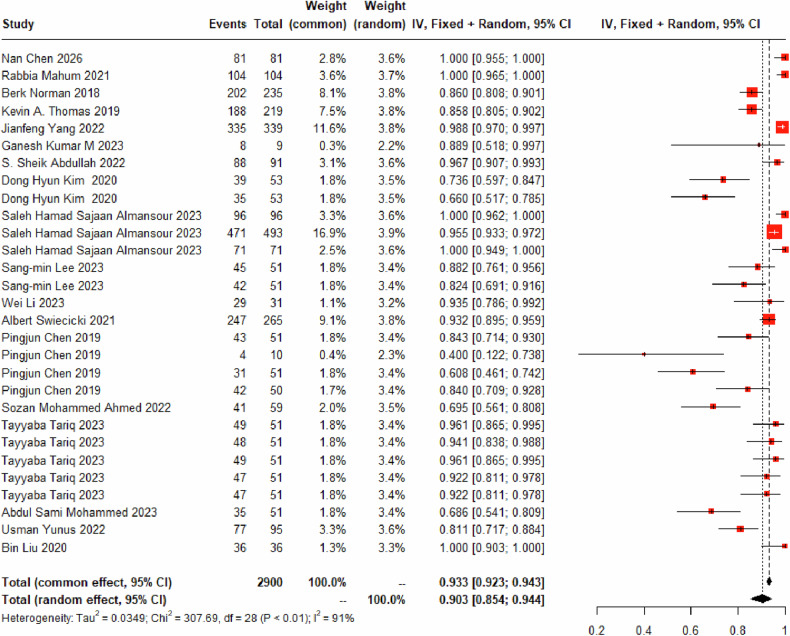
Forest plot representing the diagnostic sensitivity of K-L_4_ grade using DL based on X-ray imagery

Twenty-nine DL models were used for the identification of the K-L_4_ grade, encompassing 2630 K-L_4_ images. A random-effects model was used for data synthesis, and the pooled diagnostic sensitivity of these models for the K-L_4_ grade was determined to be 90.32% (95% CI: 85.39%–94.40%) (Fig. 6) (Table 3). The misclassification rates for K-L_4_ grade were established as follows: 0.00% (95% CI: 0.00%–0.01%) for the K-L_0_ grade (Fig. S18), 0.00% (95% CI: 0.00%–0.00%) for the K-L_1_ grade (Fig. S19), 0.04% (95% CI: 0.00%–0.38%) for the K-L_2_ grade (Fig. S20), and 8.05% (95% CI: 4.49%–12.39%) for the K-L_3_ grade (Fig. S21).

#### Publication bias

The publication bias for each level was examined, and the results showed that there was significant publication bias only for K-L4 (Egger test *p* = 0.048) (Fig. S22–Fig. 26).

## Discussion

### Summary of the main findings

DL multi-classification methods using X-ray imaging demonstrate good sensitivity (over 50%) in distinguishing K-L grades 0 to 4 for KOA. Notably, the sensitivity for K-L_4_ is exceptionally high at 90.32%, although the sensitivity rates for grades 1 and 2 are lower, indicating a need for improvement.

### Comparison with previous studies

DL is increasingly used in KOA diagnosis. Somayeh Ebrahimkhani et al [30] have found that DL models, with a Dice similarity coefficient between 85.8% to 90%, excel in knee joint cartilage segmentation compared to traditional models. Pauline Shan Qing Yeoh et al [31] have observed that DL, especially the advanced 3D Convolutional Neural Networks (3D CNNs) model [32, 33], offers more convenience and efficiency in early-stage KOA diagnosis, enabling assessment on multiple planes for a comprehensive understanding of disease progression.

Although DL shows promise in diagnosing KOA, its application in grading severity remains a subject of discussion. Most studies utilize the K-L grading system to construct classification models [2, 14, 34, 35]. Kevin Leung et al [36] developed a DL model (AUC = 0.87; 95% CI: 0.85–0.90) that outperformed the baseline K-L graded model (AUC = 0.74; 95% CI: 0.71–0.77; *p* < 0.001). Similarly, an elastic net model based on MRI by Hirvasniemi et al [37] showed superior results (AUC = 0.80) compared to a covariate model (AUC = 0.68).

However, translating these findings into clinical practice poses challenges. The complexity of KOA severity in patients goes beyond binary outcomes, necessitating multi-class results that align with clinical grading diagnoses. This systematic review, therefore, focuses on multi-classification DL methods.

The sensitivity of DL in the diagnosis of early-stage KOA (K-L_1-2_) needs to be improved. Three main factors contribute to this need. First, the K-L grading system, based on the number of osteophytes and JSN [38], often leads to radiological interpretation overlap between grades K-L_1_ and KL_2_. This overlap not only confuses radiologists but also hampers accurate parameter settings in DL models. Second, the similarities in X-ray features between K-L grades 1 and 2, like joint line height, osteophytes, subchondral sclerosis, and bone deformation [39], introduce confounding factors affecting result interpretation. Current DL models, especially CNNs, struggle with these factors, often overfitting on small datasets and failing to generalize to new data. To improve the efficacy of these models in KOA diagnosis based on K-L grading, larger sample sizes in future research are crucial. Additionally, the subjectivity of the K-L grading system [40] causes inconsistencies in interpretations across studies. Mark D. Kohn [41] highlights the evolving definitions of K-L grade 2—from “definite osteophytes with minimal joint space narrowing” [42] to “definite osteophytes with no joint space impairment” [43]. Such controversial definitions lead to biases in research results. Despite these challenges, the K-L grading system remains an essential tool in assessing KOA severity. Future research should address these diagnostic discrepancies and confounding factors to enhance the sensitivity of early-stage KOA diagnosis.

### Advantages and limitations of the study

For the first time, we explored the efficiency of DL based on X-rays for the K-L grading of KOA from a systematic review perspective. However, this study has several limitations. First, the included original studies focused on modeling variables based on radiographic images and did not discuss the actual clinical and patient situations, which could lead to inconsistencies between imaging data and clinical manifestations. Second, there was considerable heterogeneity in the meta-analysis process, which is a significant challenge for current DL. The source of this heterogeneity, seemingly related to image segmentation or image setting parameters, is difficult to further explore. Third, most of the included studies did not directly compare DL with clinical doctors, so we cannot describe whether the DL is inferior to clinical doctors.

## Conclusions

DL multi-classification methods, utilizing X-ray imaging, show promising sensitivity in differentiating K-L grades 0 to 4, with a general sensitivity rate above 50%. Specifically, these methods achieve a notably high sensitivity of 90.32% for K-L_4_. However, the sensitivity for K-L1-2 needs to be further improved.

## Supplementary information

Supplementary materials

## Abbreviations

3D CNNs: 3D Convolutional neural networks
DL: Deep learning
*I*^2^: Heterogeneity index
JSN: Joint space narrowing
K-L: Kellgren-Lawrence grading system
KOA: Knee osteoarthritis
QUADAS: Quality Assessment of Diagnostic Accuracy Studies
