# Supplementary material for: The value of deep learning-based X-ray techniques in detecting and classifying K-L grades of knee osteoarthritis: a systematic review and meta-analysis
Source: Eur Radiol. 2024 Jul 12;35(1):327–40. doi: 10.1007/s00330-024-10928-9 (PMC11631813; doi:10.1007/s00330-024-10928-9)
Supplement: Supplementary file 1 — Supplementary materials [file 330_2024_10928_MOESM1_ESM.pdf]

Supplementary materials

Table S1 Literature search strategy

1.Pubmed

**Search number**	**Query**	**Results**
**#1**	**Osteoarthritis[MeSH Terms]**	**77,203**
**#2**	**"Arthritides, Degenerative"[Title/Abstract] OR "arthritis, degenerative"[Title/Abstract] OR "arthritis, noninflammatory "[Title/Abstract] OR "Arthroses"[Title/Abstract] OR "arthrosis"[Title/Abstract] OR "Degenerative Arthritides"[Title/Abstract] OR "degenerative arthritis"[Title/Abstract] OR "degenerative joint disease"[Title/Abstract] OR "Gonarthrosis"[Title/Abstract] OR "noninflammatory arthritis"[Title/Abstract] OR "Osteoarthritides"[Title/Abstract] OR "osteoarthritis'"[Title/Abstract] OR "osteo-arthritis"[Title/Abstract] OR "Osteoarthroses"[Title/Abstract] OR "osteoarthrosis"[Title/Abstract] OR "osteo-arthrosi s"[Title/Abstract] OR "Osteoarthrosis Deformans"[Title/Abstract] OR "primary osteoarthritis"[Title/Abstract] OR "rheumatoid arthrosis"[Title/Abstract]**	**98,350**
**#3**	**("Arthritides, Degenerative"[Title/Abstract] OR "arthritis, degenerative"[Title/Abstract] OR "arthritis, noninflammatory "[Title/Abstract] OR "Arthroses"[Title/Abstract] OR "arthrosis"[Title/Abstract] OR "Degenerative Arthritides"[Title/Abstract] OR "degenerative arthritis"[Title/Abstract] OR**	**118,847**

	"degenerative joint disease"[Title/Abstract] OR "Gonarthrosis"[Title/Abstract] OR "noninflammatory arthritis"[Title/Abstract] OR "Osteoarthritides"[Title/Abstract] OR "osteoarthritis'"[Title/Abstract] OR "osteo-arthritis"[Title/Abstract] OR "Osteoarthroses"[Title/Abstract] OR "osteoarthrosis"[Title/Abstract] OR "osteo-arthrosis"[Title/Abstract] OR "Osteoarthrosis Deformans"[Title/Abstract] OR "primary osteoarthritis"[Title/Abstract] OR "rheumatoid arthrosis"[Title/Abstract]) OR (Osteoarthritis[MeSH Terms])	
#4	Deep Learning[MeSH Terms]	15,871
#5	"AlexNet"[Title/Abstract] OR "artificial intelligence"[Title/Abstract] OR "CNN"[Title/Abstract] OR "deep learning"[Title/Abstract] OR "Ensemble Learning"[Title/Abstract] OR "GoogLeNet"[Title/Abstract] OR "Hierarchical Learning"[Title/Abstract] OR "Learning, Deep"[Title/Abstract] OR "Learning, Hierarchical"[Title/Abstract] OR "Machine Learning"[Title/Abstract] OR "Neural Network"[Title/Abstract] OR "Neural Networks"[Title/Abstract] OR "ResNet"[Title/Abstract] OR "ResNet101"[Title/Abstract] OR "ResNet18"[Title/Abstract] OR "ResNet34"[Title/Abstract] OR "ResNet50"[Title/Abstract] OR "Transfer Learning"[Title/Abstract] OR "VGG11"[Title/Abstract] OR "VGG-11"[Title/Abstract] OR "VGG13"[Title/Abstract] OR "VGG-13"[Title/Abstract] OR "VGG16"[Title/Abstract] OR "VGG-16"[Title/Abstract] OR "VGG19"[Title/Abstract] OR "VGG-19"[Title/Abstract] OR	212,410

	"VGGNet"[Title/Abstract]	
#6	("AlexNet"[Title/Abstract] OR "artificial intelligence"[Title/Abstract] OR "CNN"[Title/Abstract] OR "deep learning"[Title/Abstract] OR "Ensemble Learning"[Title/Abstract] OR "GoogLeNet"[Title/Abstract] OR "Hierarchical Learning"[Title/Abstract] OR "Learning, Deep"[Title/Abstract] OR "Learning, Hierarchical"[Title/Abstract] OR "Machine Learning"[Title/Abstract] OR "Neural Network"[Title/Abstract] OR "Neural Networks"[Title/Abstract] OR "ResNet"[Title/Abstract] OR "ResNet101"[Title/Abstract] OR "ResNet18"[Title/Abstract] OR "ResNet34"[Title/Abstract] OR "ResNet50"[Title/Abstract] OR "Transfer Learning"[Title/Abstract] OR "VGG11"[Title/Abstract] OR "VGG-11"[Title/Abstract] OR "VGG13"[Title/Abstract] OR "VGG-13"[Title/Abstract] OR "VGG16"[Title/Abstract] OR "VGG-16"[Title/Abstract] OR "VGG19"[Title/Abstract] OR "VGG-19"[Title/Abstract] OR "VGGNet"[Title/Abstract]) OR (Deep Learning[MeSH Terms])	212,884
#7	(("Arthritides, Degenerative"[Title/Abstract] OR "arthritis, degenerative"[Title/Abstract] OR "arthritis, noninflammatory "[Title/Abstract] OR "Arthroses"[Title/Abstract] OR "arthrosis"[Title/Abstract] OR "Degenerative Arthritides"[Title/Abstract] OR "degenerative arthritis"[Title/Abstract] OR "degenerative joint disease"[Title/Abstract] OR "Gonarthrosis"[Title/Abstract] OR "noninflammatory arthritis"[Title/Abstract] OR "Osteoarthritides"[Title/Abstract]	645

	**OR "osteoarthritis'"[Title/Abstract] OR "osteo-arthritis"[Title/Abstract] OR "Osteoarthroses"[Title/Abstract] OR "osteoarthrosis"[Title/Abstract] OR "osteo-arthrosis"[Title/Abstract] OR "Osteoarthrosis Deformans"[Title/Abstract] OR "primary osteoarthritis"[Title/Abstract] OR "rheumatoid arthrosis"[Title/Abstract]) OR (Osteoarthritis[MeSH Terms])) AND (("AlexNet"[Title/Abstract] OR "artificial intelligence"[Title/Abstract] OR "CNN"[Title/Abstract] OR "deep learning"[Title/Abstract] OR "Ensemble Learning"[Title/Abstract] OR "GoogLeNet"[Title/Abstract] OR "Hierarchical Learning"[Title/Abstract] OR "Learning, Deep"[Title/Abstract] OR "Learning, Hierarchical"[Title/Abstract] OR "Machine Learning"[Title/Abstract] OR "Neural Network"[Title/Abstract] OR "Neural Networks"[Title/Abstract] OR "ResNet"[Title/Abstract] OR "ResNet101"[Title/Abstract] OR "ResNet18"[Title/Abstract] OR "ResNet34"[Title/Abstract] OR "ResNet50"[Title/Abstract] OR "Transfer Learning"[Title/Abstract] OR "VGG11"[Title/Abstract] OR "VGG-11"[Title/Abstract] OR "VGG13"[Title/Abstract] OR "VGG-13"[Title/Abstract] OR "VGG16"[Title/Abstract] OR "VGG-16"[Title/Abstract] OR "VGG19"[Title/Abstract] OR "VGG-19"[Title/Abstract] OR "VGGNet"[Title/Abstract]) OR (Deep Learning[MeSH Terms]))**	

2.Cochrane

Search number	Query	Results
#1	'arthritides, degenerative':ti,ab,kw OR 'arthritis, degenerative':ti,ab,kw OR 'arthritis, noninflammatory':ti,ab,kw OR 'arthroses':ti,ab,kw OR 'arthrosis':ti,ab,kw OR 'degenerative arthritides':ti,ab,kw OR 'degenerative arthritis':ti,ab,kw OR 'degenerative joint disease':ti,ab,kw OR 'gonarthrosis':ti,ab,kw OR 'noninflammatory arthritis':ti,ab,kw OR 'osteoarthritides':ti,ab,kw OR 'osteoarthritis':ti,ab,kw OR 'osteo-arthritis':ti,ab,kw OR 'osteoarthroses':ti,ab,kw OR 'osteoarthrosis':ti,ab,kw OR 'osteo-arthrosis':ti,ab,kw OR 'osteoarthrosis deformans':ti,ab,kw OR 'primary osteoarthritis':ti,ab,kw OR 'rheumatoid arthrosis':ti,ab,kw	23625
#2	MeSH descriptor: [Osteoarthritis] explode all trees	10475
#3	'alexnet':ti,ab,kw OR 'artificial intelligence':ti,ab,kw OR 'cnn':ti,ab,kw OR 'deep learning':ti,ab,kw OR 'ensemble learning':ti,ab,kw OR 'googlenet':ti,ab,kw OR 'hierarchical learning':ti,ab,kw OR 'learning, deep':ti,ab,kw OR 'learning, hierarchical':ti,ab,kw OR 'machine learning':ti,ab,kw OR 'neural network':ti,ab,kw OR 'neural networks':ti,ab,kw OR 'resnet':ti,ab,kw OR 'resnet101':ti,ab,kw OR 'resnet18':ti,ab,kw OR 'resnet34':ti,ab,kw OR 'resnet50':ti,ab,kw OR 'transfer learning':ti,ab,kw OR 'vgg11':ti,ab,kw OR 'vgg-11':ti,ab,kw OR 'vgg13':ti,ab,kw OR 'vgg-13':ti,ab,kw OR 'vgg16':ti,ab,kw OR 'vgg-16':ti,ab,kw OR 'vgg19':ti,ab,kw OR 'vgg-19':ti,ab,kw OR 'vggnet':ti,ab,kw	8908

#4	MeSH descriptor: [Deep Learning] explode all trees	272
#5	(#1 OR #2)AND(#3 OR #4)	67

3.Embase

Search number	Query	Results
#1	'osteoarthritis'/exp	163213
#2	'arthritides, degenerative':ti,ab,kw OR 'arthritis, degenerative':ti,ab,kw OR 'arthritis, noninflammatory':ti,ab,kw OR 'arthroses':ti,ab,kw OR 'arthrosis':ti,ab,kw OR 'degenerative arthritides':ti,ab,kw OR 'degenerative arthritis':ti,ab,kw OR 'degenerative joint disease':ti,ab,kw OR 'gonarthrosis':ti,ab,kw OR 'noninflammatory arthritis':ti,ab,kw OR 'osteoarthritides':ti,ab,kw OR 'osteoarthritis':ti,ab,kw OR 'osteo-arthritis':ti,ab,kw OR 'osteoarthroses':ti,ab,kw OR 'osteoarthrosis':ti,ab,kw OR 'osteo-arthrosis':ti,ab,kw OR 'osteoarthrosis deformans':ti,ab,kw OR 'primary osteoarthritis':ti,ab,kw OR 'rheumatoid arthrosis':ti,ab,kw	139574
#3	#1 OR #2	193971
#4	'deep learning'/exp	40862
#5	'alexnet':ti,ab,kw OR 'artificial intelligence':ti,ab,kw OR 'cnn':ti,ab,kw OR 'deep	250469

	learning':ti,ab,kw OR 'ensemble learning':ti,ab,kw OR 'googlenet':ti,ab,kw OR 'hierarchical learning':ti,ab,kw OR 'learning, deep':ti,ab,kw OR 'learning, hierarchical':ti,ab,kw OR 'machine learning':ti,ab,kw OR 'neural network':ti,ab,kw OR 'neural networks':ti,ab,kw OR 'resnet':ti,ab,kw OR 'resnet101':ti,ab,kw OR 'resnet18':ti,ab,kw OR 'resnet34':ti,ab,kw OR 'resnet50':ti,ab,kw OR 'transfer learning':ti,ab,kw OR 'vgg11':ti,ab,kw OR 'vgg-11':ti,ab,kw OR 'vgg13':ti,ab,kw OR 'vgg-13':ti,ab,kw OR 'vgg16':ti,ab,kw OR 'vgg-16':ti,ab,kw OR 'vgg19':ti,ab,kw OR 'vgg-19':ti,ab,kw OR 'vggnet':ti,ab,kw	
**#6**	**#4 OR #5**	**252475**
**#7**	**#3 AND #6**	**1069**

4.Web of science

**Search number**	**Query**	**Results**
**#1**	**(TS=('arthritides, degenerative':ti,ab,kw OR 'arthritis, degenerative':ti,ab,kw OR 'arthritis, noninflammatory':ti,ab,kw OR 'arthroses':ti,ab,kw OR 'arthrosis':ti,ab,kw OR 'degenerative arthritides':ti,ab,kw OR 'degenerative arthritis':ti,ab,kw OR 'degenerative joint disease':ti,ab,kw OR 'gonarthrosis':ti,ab,kw OR 'noninflammatory arthritis':ti,ab,kw OR 'osteoarthritides':ti,ab,kw OR 'osteoarthritis':ti,ab,kw OR 'osteo-arthritis':ti,ab,kw OR 'osteoarthroses':ti,ab,kw OR 'osteoarthrosis':ti,ab,kw OR 'osteo-**	**116243**

	arthrosis':ti,ab,kw OR 'osteoarthrosis deformans':ti,ab,kw OR 'primary osteoarthritis':ti,ab,kw OR 'rheumatoid arthrosis':ti,ab,kw)) OR TS=(Osteoarthritis)	
#2	(TS=('alexnet':ti,ab,kw OR 'artificial intelligence':ti,ab,kw OR 'cnn':ti,ab,kw OR 'deep learning':ti,ab,kw OR 'ensemble learning':ti,ab,kw OR 'googlenet':ti,ab,kw OR 'hierarchical learning':ti,ab,kw OR 'learning, deep':ti,ab,kw OR 'learning, hierarchical':ti,ab,kw OR 'machine learning':ti,ab,kw OR 'neural network':ti,ab,kw OR 'neural networks':ti,ab,kw OR 'resnet':ti,ab,kw OR 'resnet101':ti,ab,kw OR 'resnet18':ti,ab,kw OR 'resnet34':ti,ab,kw OR 'resnet50':ti,ab,kw OR 'transfer learning':ti,ab,kw OR 'vgg11':ti,ab,kw OR 'vgg-11':ti,ab,kw OR 'vgg13':ti,ab,kw OR 'vgg-13':ti,ab,kw OR 'vgg16':ti,ab,kw OR 'vgg-16':ti,ab,kw OR 'vgg19':ti,ab,kw OR 'vgg-19':ti,ab,kw OR 'vggnet':ti,ab,kw)) OR TS=(deep learning)	270003
#3	#1 AND #2	353

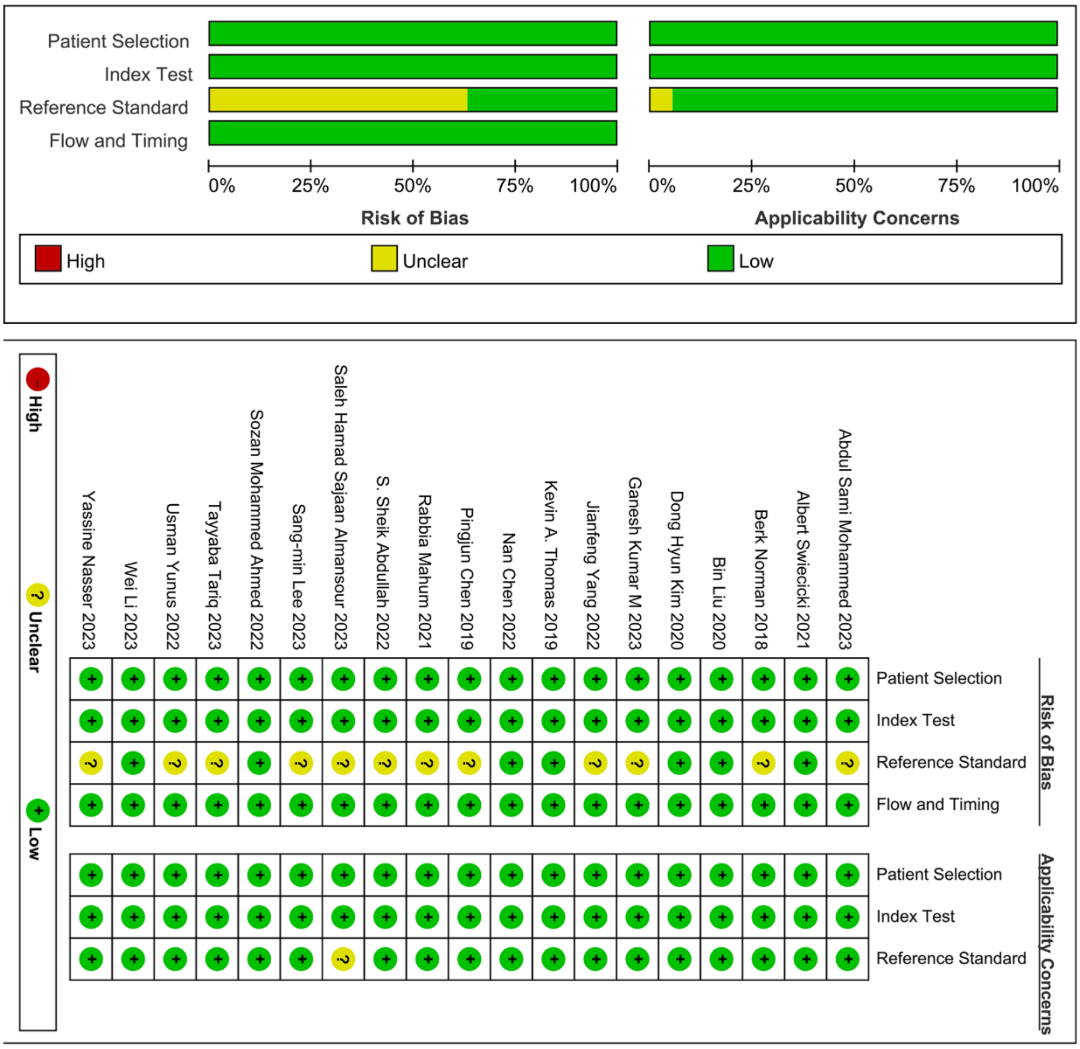

Figure S1 Risk of bias diagram

Study	Events	Total	Weight (common)	Weight (random)	IV, Fixed + Random, 95% CI
Yassine Nasser 2023	436	2313	10.3%	3.5%	0.188 [0.173; 0.205]
Yassine Nasser 2023	294	2313	10.3%	3.5%	0.127 [0.114; 0.141]
Nan Chen 2022	31	182	0.8%	3.4%	0.170 [0.119; 0.233]
Rabbia Mahum 2021	15	52	0.2%	3.1%	0.288 [0.171; 0.431]
Kevin A. Thomas 2019	127	1441	6.4%	3.5%	0.088 [0.074; 0.104]
Ganesh Kumar M 2023	14	49	0.2%	3.1%	0.286 [0.166; 0.433]
S. Sheik Abdullah 2022	0	159	0.7%	3.4%	0.000 [0.000; 0.023]
Dong Hyun Kim 2020	12	53	0.2%	3.1%	0.226 [0.123; 0.362]
Dong Hyun Kim 2020	30	74	0.3%	3.2%	0.405 [0.293; 0.526]
Saleh Hamad Sajaan Almansour 2023	229	575	2.6%	3.5%	0.398 [0.358; 0.440]
Saleh Hamad Sajaan Almansour 2023	1	493	2.2%	3.5%	0.002 [0.000; 0.011]
Saleh Hamad Sajaan Almansour 2023	22	984	4.4%	3.5%	0.022 [0.014; 0.034]
Sang-min Lee 2023	25	639	2.9%	3.5%	0.039 [0.025; 0.057]
Sang-min Lee 2023	55	639	2.9%	3.5%	0.086 [0.065; 0.111]
Wei Li 2023	3	399	1.8%	3.5%	0.008 [0.002; 0.022]
Albert Swiecicki 2021	315	1283	5.7%	3.5%	0.246 [0.222; 0.270]
Pingjun Chen 2019	43	639	2.9%	3.5%	0.067 [0.049; 0.090]
Pingjun Chen 2019	42	639	2.9%	3.5%	0.066 [0.048; 0.088]
Pingjun Chen 2019	52	639	2.9%	3.5%	0.081 [0.061; 0.105]
Pingjun Chen 2019	105	639	2.9%	3.5%	0.164 [0.136; 0.195]
Sozan Mohammed Ahmed 2022	41	772	3.4%	3.5%	0.053 [0.038; 0.071]
Tayyaba Tariq 2023	3	639	2.9%	3.5%	0.005 [0.001; 0.014]
Tayyaba Tariq 2023	3	639	2.9%	3.5%	0.005 [0.001; 0.014]
Tayyaba Tariq 2023	21	639	2.9%	3.5%	0.033 [0.020; 0.050]
Tayyaba Tariq 2023	5	639	2.9%	3.5%	0.008 [0.003; 0.018]
Tayyaba Tariq 2023	1	639	2.9%	3.5%	0.002 [0.000; 0.009]
Abdul Sami Mohammed 2023	26	639	2.9%	3.5%	0.041 [0.027; 0.059]
Usman Yunus 2022	96	3382	15.1%	3.5%	0.028 [0.023; 0.035]
Bin Liu 2020	2	196	0.9%	3.4%	0.010 [0.001; 0.036]
**Total (common effect, 95% CI)**		**22388**	**100.0%**	--	**0.071 [0.068; 0.075]**
**Total (random effect, 95% CI)**			--	**100.0%**	**0.077 [0.043; 0.119]**

Heterogeneity: $Tau^2 = 0.0362$; $Chi^2 = 2110.26$, df = 28 (P = 0); $I^2 = 99\%$

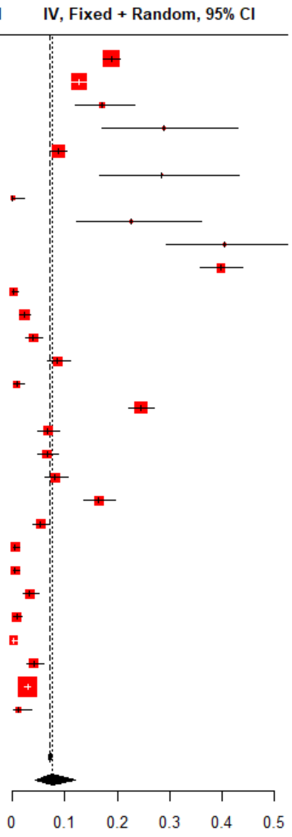

Figure S2: Forest Plot for the Misdiagnosis Rate of K-L_0 Diagnosis by DL Based on X-ray, Misclassified as K-L_1 Grade

Study	Events	Total	Weight (common)	Weight (random)	IV, Fixed + Random, 95% CI
Yassine Nasser 2023	44	2313	10.3%	3.7%	0.019 [0.014; 0.025]
Yassine Nasser 2023	58	2313	10.3%	3.7%	0.025 [0.019; 0.032]
Nan Chen 2022	10	182	0.8%	3.3%	0.055 [0.027; 0.099]
Rabbia Mahum 2021	12	52	0.2%	2.7%	0.231 [0.125; 0.368]
Kevin A. Thomas 2019	65	1441	6.4%	3.6%	0.045 [0.035; 0.057]
Ganesh Kumar M 2023	5	49	0.2%	2.7%	0.102 [0.034; 0.222]
S. Sheik Abdullah 2022	0	159	0.7%	3.3%	0.000 [0.000; 0.023]
Dong Hyun Kim 2020	5	53	0.2%	2.7%	0.094 [0.031; 0.207]
Dong Hyun Kim 2020	4	74	0.3%	2.9%	0.054 [0.015; 0.133]
Saleh Hamad Sajaan Almansour 2023	139	575	2.6%	3.6%	0.242 [0.207; 0.279]
Saleh Hamad Sajaan Almansour 2023	35	493	2.2%	3.5%	0.071 [0.050; 0.097]
Saleh Hamad Sajaan Almansour 2023	8	984	4.4%	3.6%	0.008 [0.004; 0.016]
Sang-min Lee 2023	30	639	2.9%	3.6%	0.047 [0.032; 0.066]
Sang-min Lee 2023	22	639	2.9%	3.6%	0.034 [0.022; 0.052]
Wei Li 2023	1	399	1.8%	3.5%	0.003 [0.000; 0.014]
Albert Swiecicki 2021	36	1283	5.7%	3.6%	0.028 [0.020; 0.039]
Pingjun Chen 2019	39	639	2.9%	3.6%	0.061 [0.044; 0.082]
Pingjun Chen 2019	19	639	2.9%	3.6%	0.030 [0.018; 0.046]
Pingjun Chen 2019	19	639	2.9%	3.6%	0.030 [0.018; 0.046]
Pingjun Chen 2019	27	639	2.9%	3.6%	0.042 [0.028; 0.061]
Sozan Mohammed Ahmed 2022	57	772	3.4%	3.6%	0.074 [0.056; 0.095]
Tayyaba Tariq 2023	4	639	2.9%	3.6%	0.006 [0.002; 0.016]
Tayyaba Tariq 2023	0	639	2.9%	3.6%	0.000 [0.000; 0.006]
Tayyaba Tariq 2023	3	639	2.9%	3.6%	0.005 [0.001; 0.014]
Tayyaba Tariq 2023	2	639	2.9%	3.6%	0.003 [0.000; 0.011]
Tayyaba Tariq 2023	1	639	2.9%	3.6%	0.002 [0.000; 0.009]
Abdul Sami Mohammed 2023	43	639	2.9%	3.6%	0.067 [0.049; 0.090]
Usman Yunus 2022	79	3382	15.1%	3.7%	0.023 [0.019; 0.029]
Bin Liu 2020	18	196	0.9%	3.4%	0.092 [0.055; 0.141]
**Total (common effect, 95% CI)**		**22388**	**100.0%**	--	**0.027 [0.025; 0.029]**
**Total (random effect, 95% CI)**			--	**100.0%**	**0.036 [0.021; 0.054]**

Heterogeneity: $Tau^2 = 0.0133$; $Chi^2 = 681.39$, df = 28 ($P < 0.01$); $I^2 = 96\%$

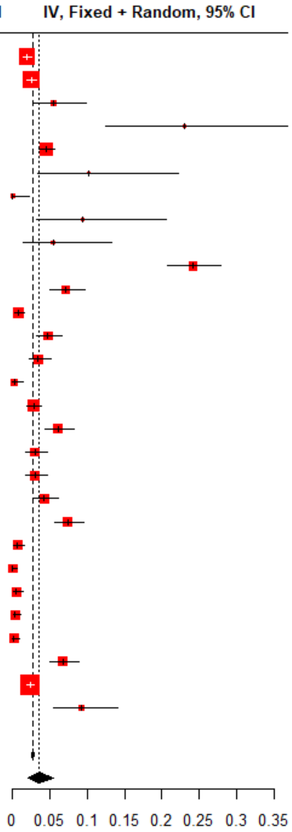

Figure S3: Forest Plot for the Misdiagnosis Rate of K-L_0 Diagnosis by DL Based on X-ray, Misclassified as K-L_2 Grade

Study	Events	Total	Weight (common)	Weight (random)	IV, Fixed + Random, 95% CI
Yassine Nasser 2023	.	2313	0.0%	0.0%	
Yassine Nasser 2023	.	2313	0.0%	0.0%	
Nan Chen 2022	0	182	1.0%	3.3%	0.000 [0.000; 0.020]
Rabbia Mahum 2021	9	52	0.3%	2.0%	0.173 [0.082; 0.303]
Kevin A. Thomas 2019	2	1441	8.1%	4.3%	0.001 [0.000; 0.005]
Ganesh Kumar M 2023	0	49	0.3%	1.9%	0.000 [0.000; 0.073]
S. Sheik Abdullah 2022	0	159	0.9%	3.2%	0.000 [0.000; 0.023]
Dong Hyun Kim 2020	0	53	0.3%	2.0%	0.000 [0.000; 0.067]
Dong Hyun Kim 2020	2	74	0.4%	2.4%	0.027 [0.003; 0.094]
Saleh Hamad Sajaan Almansour 2023	34	575	3.2%	4.0%	0.059 [0.041; 0.082]
Saleh Hamad Sajaan Almansour 2023	0	493	2.8%	4.0%	0.000 [0.000; 0.007]
Saleh Hamad Sajaan Almansour 2023	0	984	5.5%	4.2%	0.000 [0.000; 0.004]
Sang-min Lee 2023	1	639	3.6%	4.1%	0.002 [0.000; 0.009]
Sang-min Lee 2023	0	639	3.6%	4.1%	0.000 [0.000; 0.006]
Wei Li 2023	0	399	2.2%	3.8%	0.000 [0.000; 0.009]
Albert Swiecicki 2021	1	1283	7.2%	4.3%	0.001 [0.000; 0.004]
Pingjun Chen 2019	1	639	3.6%	4.1%	0.002 [0.000; 0.009]
Pingjun Chen 2019	0	639	3.6%	4.1%	0.000 [0.000; 0.006]
Pingjun Chen 2019	0	639	3.6%	4.1%	0.000 [0.000; 0.006]
Pingjun Chen 2019	1	639	3.6%	4.1%	0.002 [0.000; 0.009]
Sozan Mohammed Ahmed 2022	5	772	4.3%	4.1%	0.006 [0.002; 0.015]
Tayyaba Tariq 2023	0	639	3.6%	4.1%	0.000 [0.000; 0.006]
Tayyaba Tariq 2023	0	639	3.6%	4.1%	0.000 [0.000; 0.006]
Tayyaba Tariq 2023	0	639	3.6%	4.1%	0.000 [0.000; 0.006]
Tayyaba Tariq 2023	0	639	3.6%	4.1%	0.000 [0.000; 0.006]
Tayyaba Tariq 2023	0	639	3.6%	4.1%	0.000 [0.000; 0.006]
Abdul Sami Mohammed 2023	10	639	3.6%	4.1%	0.016 [0.008; 0.029]
Usman Yunus 2022	8	3382	19.0%	4.4%	0.002 [0.001; 0.005]
Bin Liu 2020	0	196	1.1%	3.4%	0.000 [0.000; 0.019]
**Total (common effect, 95% CI)**		**22388**	**100.0%**	--	**0.001 [0.000; 0.001]**
**Total (random effect, 95% CI)**			--	**100.0%**	**0.002 [0.000; 0.005]**

Heterogeneity: $Tau^2 = 0.0038$; $Chi^2 = 173.49$, df = 26 ($P < 0.01$); $I^2 = 85\%$

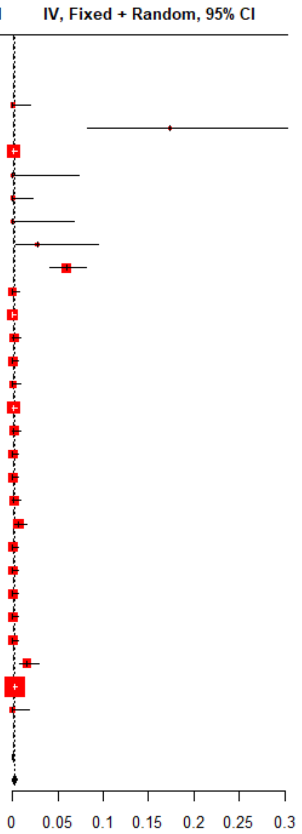

Figure S4: Forest Plot for the Misdiagnosis Rate of K-L$_0$ Diagnosis by DL Based on X-ray, Misclassified as K-L$_3$ Grade

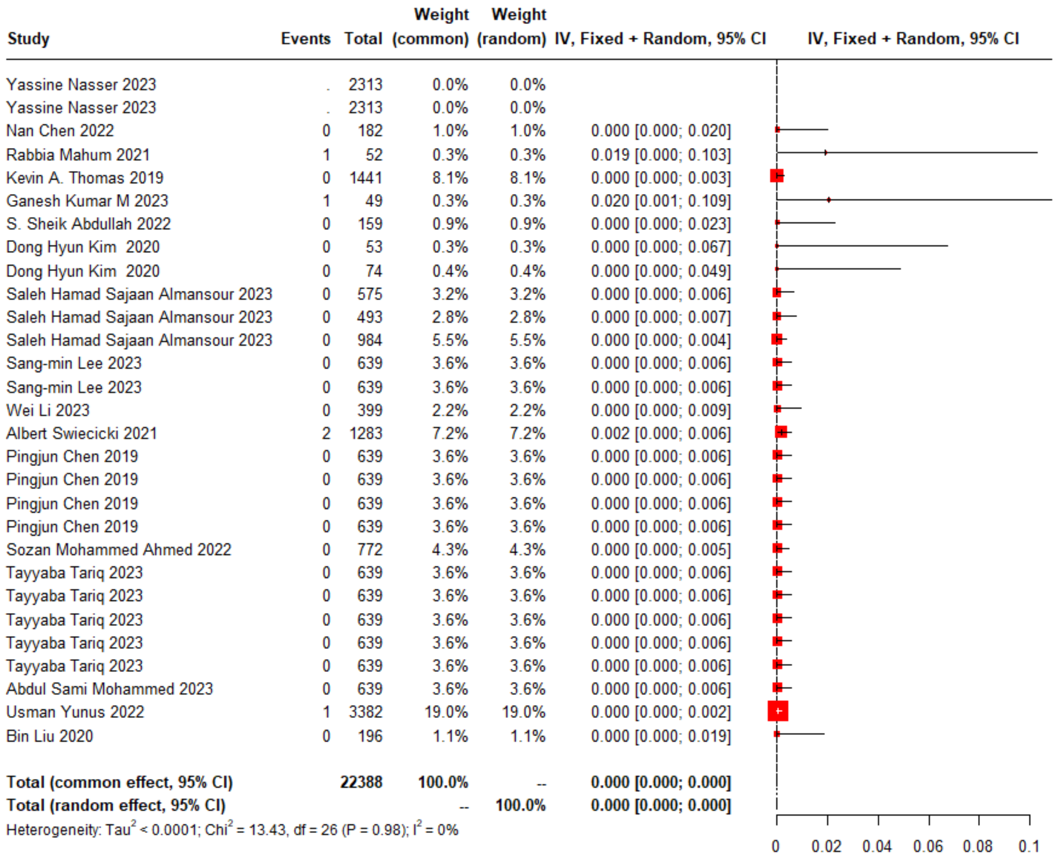

Figure S5: Forest Plot for the Misdiagnosis Rate of K-L_0 Diagnosis by DL Based on X-ray, Misclassified as K-L_4 Grade

Study	Events	Total	Weight (common)	Weight (random)	IV, Fixed + Random, 95% CI
Yassine Nasser 2023	490	1071	10.3%	3.4%	0.458 [0.427; 0.488]
Yassine Nasser 2023	523	1071	10.3%	3.4%	0.488 [0.458; 0.519]
Nan Chen 2023	30	101	1.0%	3.3%	0.297 [0.210; 0.396]
Rabbia Mahum 2021	6	81	0.8%	3.3%	0.074 [0.028; 0.154]
Berk Norman 2018	.	3049	0.0%	0.0%	
Kevin A. Thomas 2019	324	656	6.3%	3.4%	0.494 [0.455; 0.533]
Jianfeng Yang 2022	0	58	0.6%	3.2%	0.000 [0.000; 0.062]
Ganesh Kumar M 2023	13	44	0.4%	3.2%	0.295 [0.168; 0.452]
S. Sheik Abdullah 2022	1	183	1.8%	3.3%	0.005 [0.000; 0.030]
Dong Hyun Kim 2020	17	64	0.6%	3.2%	0.266 [0.163; 0.391]
Dong Hyun Kim 2020	8	117	1.1%	3.3%	0.068 [0.030; 0.130]
Saleh Hamad Sajaan Almansour 2023	304	700	6.7%	3.4%	0.434 [0.397; 0.472]
Saleh Hamad Sajaan Almansour 2023	0	492	4.7%	3.4%	0.000 [0.000; 0.007]
Saleh Hamad Sajaan Almansour 2023	0	458	4.4%	3.4%	0.000 [0.000; 0.008]
Sang-min Lee 2023	104	296	2.9%	3.4%	0.351 [0.297; 0.409]
Sang-min Lee 2023	134	296	2.9%	3.4%	0.453 [0.395; 0.511]
Wei Li 2023	8	213	2.1%	3.3%	0.038 [0.016; 0.073]
Albert Swiecicki 2021	126	526	5.1%	3.4%	0.240 [0.204; 0.278]
Pingjun Chen 2019	131	296	2.9%	3.4%	0.443 [0.385; 0.501]
Pingjun Chen 2019	159	296	2.9%	3.4%	0.537 [0.479; 0.595]
Pingjun Chen 2019	131	296	2.9%	3.4%	0.443 [0.385; 0.501]
Pingjun Chen 2019	101	296	2.9%	3.4%	0.341 [0.287; 0.398]
Sozan Mohammed Ahmed 2022	219	354	3.4%	3.4%	0.619 [0.566; 0.669]
Tayyaba Tariq 2023	19	296	2.9%	3.4%	0.064 [0.039; 0.098]
Tayyaba Tariq 2023	11	296	2.9%	3.4%	0.037 [0.019; 0.066]
Tayyaba Tariq 2023	14	296	2.9%	3.4%	0.047 [0.026; 0.078]
Tayyaba Tariq 2023	13	296	2.9%	3.4%	0.044 [0.024; 0.074]
Tayyaba Tariq 2023	11	296	2.9%	3.4%	0.037 [0.019; 0.066]
Abdul Sami Mohammed 2023	132	296	2.9%	3.4%	0.446 [0.388; 0.505]
Usman Yunus 2022	79	571	5.5%	3.4%	0.138 [0.111; 0.169]
Bin Liu 2020	4	54	0.5%	3.2%	0.074 [0.021; 0.179]
**Total (common effect, 95% CI)**		**13415**	**100.0%**	--	**0.257 [0.248; 0.265]**
**Total (random effect, 95% CI)**		--	--	**100.0%**	**0.198 [0.125; 0.283]**

Heterogeneity: $Tau^2 = 0.0752$; $Chi^2 = 3187.24$, df = 29 (P = 0); $I^2 = 99\%$

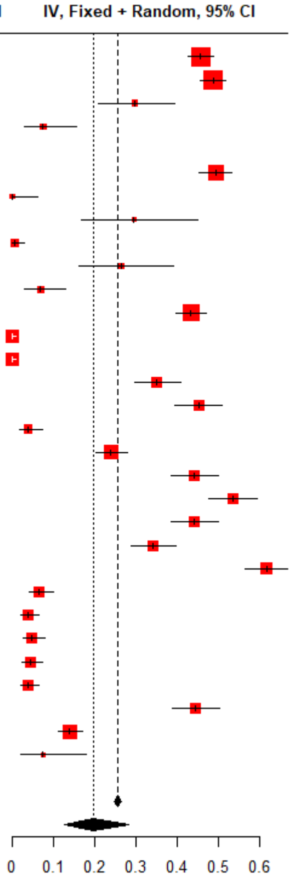

Figure S6: Forest Plot for the Misdiagnosis Rate of K-L1 Diagnosis Using DL Based on X-ray, Misclassified as K-L0 Grade

Study	Events	Total	Weight (common)	Weight (random)	IV, Fixed + Random, 95% CI
Yassine Nasser 2023	122	1071	8.0%	3.3%	0.114 [0.095; 0.134]
Yassine Nasser 2023	138	1071	8.0%	3.3%	0.129 [0.109; 0.150]
Nan Chen 2023	25	101	0.8%	3.1%	0.248 [0.167; 0.343]
Rabbia Mahum 2021	0	81	0.6%	3.1%	0.000 [0.000; 0.045]
Berk Norman 2018	475	3049	22.7%	3.3%	0.156 [0.143; 0.169]
Kevin A. Thomas 2019	146	656	4.9%	3.3%	0.223 [0.191; 0.256]
Jianfeng Yang 2022	3	58	0.4%	3.0%	0.052 [0.011; 0.144]
Ganesh Kumar M 2023	6	44	0.3%	2.9%	0.136 [0.052; 0.274]
S. Sheik Abdullah 2022	2	183	1.4%	3.2%	0.011 [0.001; 0.039]
Dong Hyun Kim 2020	7	64	0.5%	3.0%	0.109 [0.045; 0.212]
Dong Hyun Kim 2020	18	117	0.9%	3.2%	0.154 [0.094; 0.232]
Saleh Hamad Sajaan Almansour 2023	184	700	5.2%	3.3%	0.263 [0.231; 0.297]
Saleh Hamad Sajaan Almansour 2023	0	492	3.7%	3.3%	0.000 [0.000; 0.007]
Saleh Hamad Sajaan Almansour 2023	3	458	3.4%	3.3%	0.007 [0.001; 0.019]
Sang-min Lee 2023	44	296	2.2%	3.3%	0.149 [0.110; 0.194]
Sang-min Lee 2023	66	296	2.2%	3.3%	0.223 [0.177; 0.275]
Wei Li 2023	9	213	1.6%	3.2%	0.042 [0.020; 0.079]
Albert Swiecicki 2021	104	526	3.9%	3.3%	0.198 [0.165; 0.234]
Pingjun Chen 2019	108	296	2.2%	3.3%	0.365 [0.310; 0.423]
Pingjun Chen 2019	66	296	2.2%	3.3%	0.223 [0.177; 0.275]
Pingjun Chen 2019	108	296	2.2%	3.3%	0.365 [0.310; 0.423]
Pingjun Chen 2019	74	296	2.2%	3.3%	0.250 [0.202; 0.303]
Sozan Mohammed Ahmed 2022	81	354	2.6%	3.3%	0.229 [0.186; 0.276]
Tayyaba Tariq 2023	15	296	2.2%	3.3%	0.051 [0.029; 0.082]
Tayyaba Tariq 2023	3	296	2.2%	3.3%	0.010 [0.002; 0.029]
Tayyaba Tariq 2023	6	296	2.2%	3.3%	0.020 [0.007; 0.044]
Tayyaba Tariq 2023	14	296	2.2%	3.3%	0.047 [0.026; 0.078]
Tayyaba Tariq 2023	5	296	2.2%	3.3%	0.017 [0.006; 0.039]
Abdul Sami Mohammed 2023	53	296	2.2%	3.3%	0.179 [0.137; 0.228]
Usman Yunus 2022	4	571	4.3%	3.3%	0.007 [0.002; 0.018]
Bin Liu 2020	18	54	0.4%	3.0%	0.333 [0.211; 0.475]
**Total (common effect, 95% CI)**		**13415**	**100.0%**	--	**0.121 [0.115; 0.127]**
**Total (random effect, 95% CI)**			--	**100.0%**	**0.112 [0.074; 0.158]**

Heterogeneity: $Tau^2 = 0.0339$; $Chi^2 = 1373.49$, df = 30 ($P < 0.01$); $I^2 = 98\%$

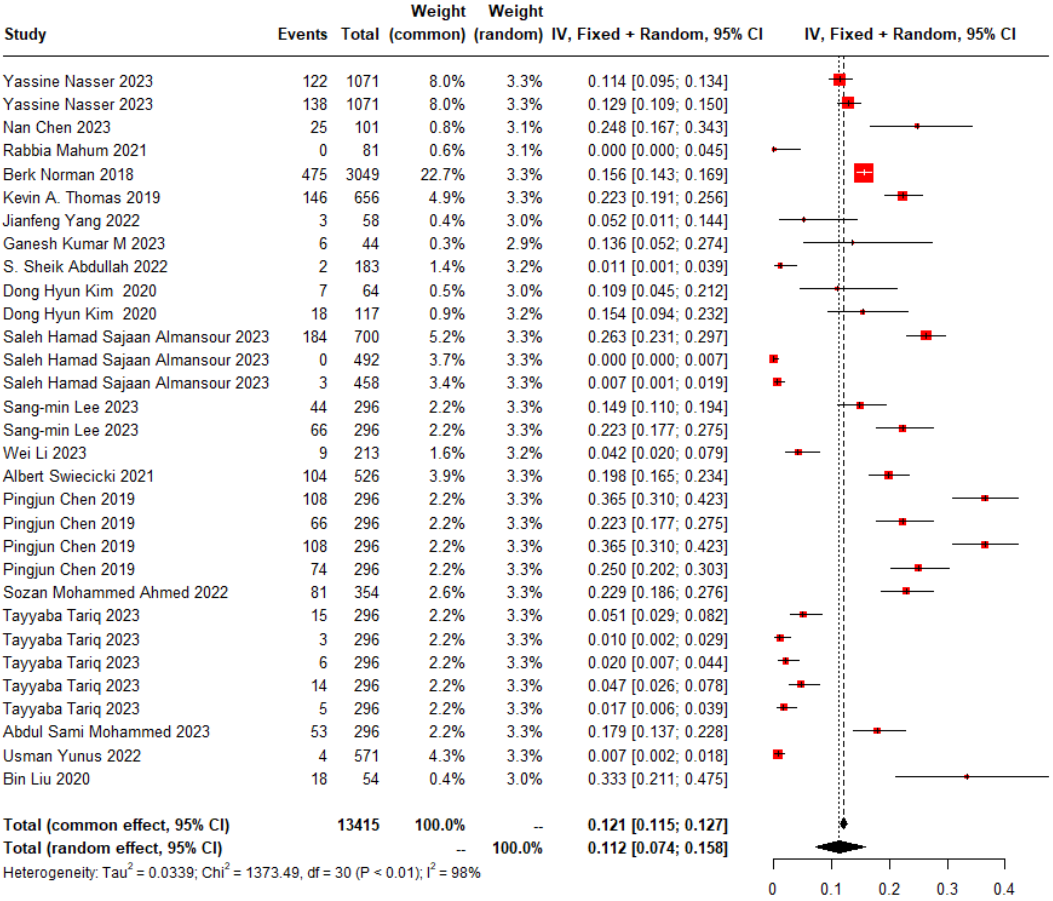

Figure S7: Forest Plot for the Misdiagnosis Rate of K-L_1 Diagnosis Using DL Based on X-ray, Misclassified as K-L_2 Grade

Study	Events	Total	Weight (common)	Weight (random)	IV, Fixed + Random, 95% CI
Yassine Nasser 2023	.	1071	0.0%	0.0%	
Yassine Nasser 2023	.	1071	0.0%	0.0%	
Nan Chen 2023	2	101	0.9%	2.7%	0.020 [0.002; 0.070]
Rabbia Mahum 2021	0	81	0.7%	2.5%	0.000 [0.000; 0.045]
Berk Norman 2018	17	3049	27.0%	4.4%	0.006 [0.003; 0.009]
Kevin A. Thomas 2019	9	656	5.8%	4.1%	0.014 [0.006; 0.026]
Jianfeng Yang 2022	2	58	0.5%	2.1%	0.034 [0.004; 0.119]
Ganesh Kumar M 2023	2	44	0.4%	1.8%	0.045 [0.006; 0.155]
S. Sheik Abdullah 2022	0	183	1.6%	3.3%	0.000 [0.000; 0.020]
Dong Hyun Kim 2020	4	64	0.6%	2.2%	0.062 [0.017; 0.152]
Dong Hyun Kim 2020	6	117	1.0%	2.9%	0.051 [0.019; 0.108]
Saleh Hamad Sajaan Almansour 2023	49	700	6.2%	4.1%	0.070 [0.052; 0.091]
Saleh Hamad Sajaan Almansour 2023	20	492	4.4%	4.0%	0.041 [0.025; 0.062]
Saleh Hamad Sajaan Almansour 2023	0	458	4.1%	4.0%	0.000 [0.000; 0.008]
Sang-min Lee 2023	0	296	2.6%	3.7%	0.000 [0.000; 0.012]
Sang-min Lee 2023	1	296	2.6%	3.7%	0.003 [0.000; 0.019]
Wei Li 2023	2	213	1.9%	3.5%	0.009 [0.001; 0.034]
Albert Swiecicki 2021	1	526	4.7%	4.0%	0.002 [0.000; 0.011]
Pingjun Chen 2019	5	296	2.6%	3.7%	0.017 [0.006; 0.039]
Pingjun Chen 2019	8	296	2.6%	3.7%	0.027 [0.012; 0.053]
Pingjun Chen 2019	5	296	2.6%	3.7%	0.017 [0.006; 0.039]
Pingjun Chen 2019	7	296	2.6%	3.7%	0.024 [0.010; 0.048]
Sozan Mohammed Ahmed 2022	10	354	3.1%	3.8%	0.028 [0.014; 0.051]
Tayyaba Tariq 2023	1	296	2.6%	3.7%	0.003 [0.000; 0.019]
Tayyaba Tariq 2023	1	296	2.6%	3.7%	0.003 [0.000; 0.019]
Tayyaba Tariq 2023	1	296	2.6%	3.7%	0.003 [0.000; 0.019]
Tayyaba Tariq 2023	1	296	2.6%	3.7%	0.003 [0.000; 0.019]
Tayyaba Tariq 2023	2	296	2.6%	3.7%	0.007 [0.001; 0.024]
Abdul Sami Mohammed 2023	6	296	2.6%	3.7%	0.020 [0.007; 0.044]
Usman Yunus 2022	1	571	5.1%	4.1%	0.002 [0.000; 0.010]
Bin Liu 2020	0	54	0.5%	2.0%	0.000 [0.000; 0.066]
**Total (common effect, 95% CI)**		**13415**	**100.0%**	--	**0.009 [0.007; 0.011]**
**Total (random effect, 95% CI)**			--	**100.0%**	**0.011 [0.006; 0.018]**

Heterogeneity: $Tau^2 = 0.0038$; $Chi^2 = 190.54$, df = 28 ($P < 0.01$); $I^2 = 85\%$

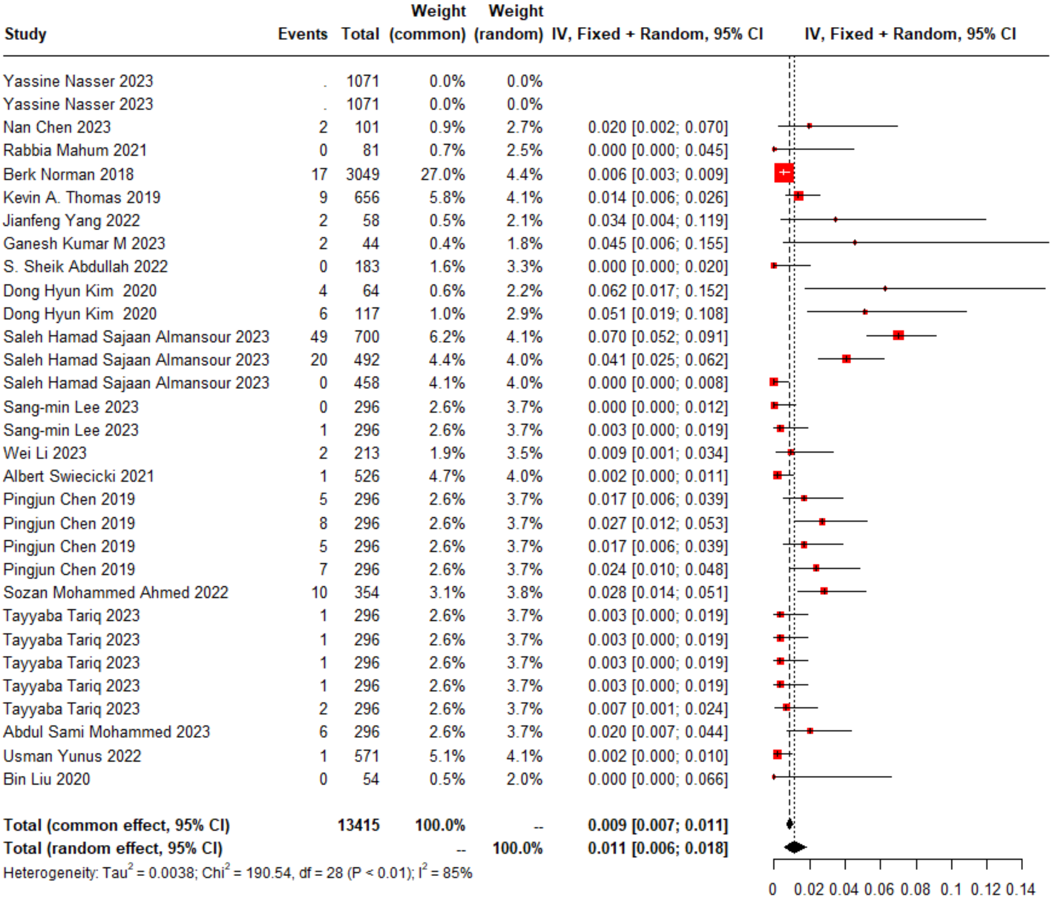

Figure S8: Forest Plot for the Misdiagnosis Rate of K-L_1 Diagnosis Using DL Based on X-ray, Misclassified as K-L_3 Grade

Study	Events	Total	Weight (common)	Weight (random)	IV, Fixed + Random, 95% CI
Yassine Nasser 2023	.	1071	0.0%	0.0%	
Yassine Nasser 2023	.	1071	0.0%	0.0%	
Nan Chen 2023	0	101	0.9%	0.9%	0.000 [0.000; 0.036]
Rabbia Mahum 2021	0	81	0.7%	0.7%	0.000 [0.000; 0.045]
Berk Norman 2018	4	3049	27.0%	26.8%	0.001 [0.000; 0.003]
Kevin A. Thomas 2019	0	656	5.8%	5.8%	0.000 [0.000; 0.006]
Jianfeng Yang 2022	0	58	0.5%	0.5%	0.000 [0.000; 0.062]
Ganesh Kumar M 2023	0	44	0.4%	0.4%	0.000 [0.000; 0.080]
S. Sheik Abdullah 2022	0	183	1.6%	1.6%	0.000 [0.000; 0.020]
Dong Hyun Kim 2020	0	64	0.6%	0.6%	0.000 [0.000; 0.056]
Dong Hyun Kim 2020	1	117	1.0%	1.0%	0.009 [0.000; 0.047]
Saleh Hamad Sajaan Almansour 2023	0	700	6.2%	6.2%	0.000 [0.000; 0.005]
Saleh Hamad Sajaan Almansour 2023	2	492	4.4%	4.4%	0.004 [0.000; 0.015]
Saleh Hamad Sajaan Almansour 2023	2	458	4.1%	4.1%	0.004 [0.001; 0.016]
Sang-min Lee 2023	0	296	2.6%	2.6%	0.000 [0.000; 0.012]
Sang-min Lee 2023	0	296	2.6%	2.6%	0.000 [0.000; 0.012]
Wei Li 2023	0	213	1.9%	1.9%	0.000 [0.000; 0.017]
Albert Swiecicki 2021	1	526	4.7%	4.7%	0.002 [0.000; 0.011]
Pingjun Chen 2019	0	296	2.6%	2.6%	0.000 [0.000; 0.012]
Pingjun Chen 2019	0	296	2.6%	2.6%	0.000 [0.000; 0.012]
Pingjun Chen 2019	0	296	2.6%	2.6%	0.000 [0.000; 0.012]
Pingjun Chen 2019	0	296	2.6%	2.6%	0.000 [0.000; 0.012]
Sozan Mohammed Ahmed 2022	0	354	3.1%	3.1%	0.000 [0.000; 0.010]
Tayyaba Tariq 2023	0	296	2.6%	2.6%	0.000 [0.000; 0.012]
Tayyaba Tariq 2023	0	296	2.6%	2.6%	0.000 [0.000; 0.012]
Tayyaba Tariq 2023	0	296	2.6%	2.6%	0.000 [0.000; 0.012]
Tayyaba Tariq 2023	0	296	2.6%	2.6%	0.000 [0.000; 0.012]
Tayyaba Tariq 2023	0	296	2.6%	2.6%	0.000 [0.000; 0.012]
Abdul Sami Mohammed 2023	0	296	2.6%	2.6%	0.000 [0.000; 0.012]
Usman Yunus 2022	0	571	5.1%	5.1%	0.000 [0.000; 0.006]
Bin Liu 2020	2	54	0.5%	0.5%	0.037 [0.005; 0.127]
**Total (common effect, 95% CI)**		**13415**	**100.0%**	--	**0.000 [0.000; 0.000]**
**Total (random effect, 95% CI)**			--	**100.0%**	**0.000 [0.000; 0.000]**

Heterogeneity: $Tau^2 < 0.0001$; $Chi^2 = 18.80$, df = 28 ($P = 0.90$); $I^2 = 0\%$

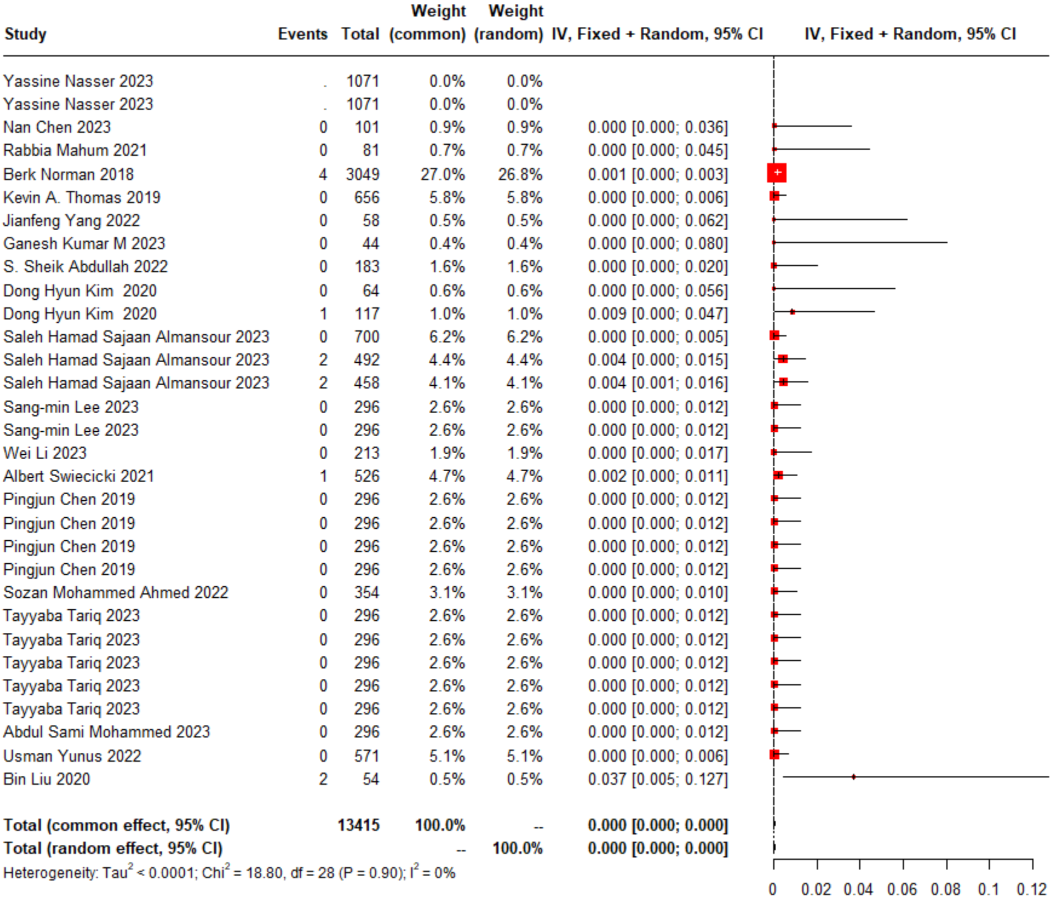

Figure S9: Forest Plot for the Misdiagnosis Rate of K-L$_1$ Diagnosis Using DL Based on X-ray, Misclassified as K-L$_4$ Grade

Study	Events	Total	Weight (common)	Weight (random)	IV, Fixed + Random, 95% CI
Yassine Nasser 2023	151	1545	10.8%	3.5%	0.098 [0.083; 0.114]
Yassine Nasser 2023	165	1545	10.8%	3.5%	0.107 [0.092; 0.123]
Nan Chen 2024	7	128	0.9%	3.2%	0.055 [0.022; 0.109]
Rabbia Mahum 2021	2	85	0.6%	3.1%	0.024 [0.003; 0.082]
Berk Norman 2018	.	1306	0.0%	0.0%	
Kevin A. Thomas 2019	136	1114	7.8%	3.4%	0.122 [0.103; 0.143]
Jianfeng Yang 2022	0	137	1.0%	3.2%	0.000 [0.000; 0.027]
Ganesh Kumar M 2023	3	33	0.2%	2.7%	0.091 [0.019; 0.243]
S. Sheik Abdullah 2022	0	108	0.8%	3.2%	0.000 [0.000; 0.034]
Dong Hyun Kim 2020	9	99	0.7%	3.2%	0.091 [0.042; 0.166]
Dong Hyun Kim 2020	6	125	0.9%	3.2%	0.048 [0.018; 0.102]
Saleh Hamad Sajaan Almansour 2023	62	609	4.3%	3.4%	0.102 [0.079; 0.129]
Saleh Hamad Sajaan Almansour 2023	172	490	3.4%	3.4%	0.351 [0.309; 0.395]
Saleh Hamad Sajaan Almansour 2023	22	611	4.3%	3.4%	0.036 [0.023; 0.054]
Sang-min Lee 2023	56	447	3.1%	3.4%	0.125 [0.096; 0.160]
Sang-min Lee 2023	39	447	3.1%	3.4%	0.087 [0.063; 0.117]
Wei Li 2023	0	126	0.9%	3.2%	0.000 [0.000; 0.029]
Albert Swiecicki 2021	5	588	4.1%	3.4%	0.009 [0.003; 0.020]
Pingjun Chen 2019	41	447	3.1%	3.4%	0.092 [0.067; 0.122]
Pingjun Chen 2019	61	447	3.1%	3.4%	0.136 [0.106; 0.172]
Pingjun Chen 2019	45	447	3.1%	3.4%	0.101 [0.074; 0.132]
Pingjun Chen 2019	24	447	3.1%	3.4%	0.054 [0.035; 0.079]
Sozan Mohammed Ahmed 2022	168	516	3.6%	3.4%	0.326 [0.285; 0.368]
Tayyaba Tariq 2023	4	447	3.1%	3.4%	0.009 [0.002; 0.023]
Tayyaba Tariq 2023	9	447	3.1%	3.4%	0.020 [0.009; 0.038]
Tayyaba Tariq 2023	5	447	3.1%	3.4%	0.011 [0.004; 0.026]
Tayyaba Tariq 2023	5	447	3.1%	3.4%	0.011 [0.004; 0.026]
Tayyaba Tariq 2023	3	447	3.1%	3.4%	0.007 [0.001; 0.019]
Abdul Sami Mohammed 2023	114	447	3.1%	3.4%	0.255 [0.215; 0.298]
Usman Yunus 2022	145	850	5.9%	3.4%	0.171 [0.146; 0.198]
Bin Liu 2020	24	218	1.5%	3.3%	0.110 [0.072; 0.159]
**Total (common effect, 95% CI)**		**15597**	**100.0%**	--	**0.087 [0.082; 0.091]**
**Total (random effect, 95% CI)**			--	**100.0%**	**0.069 [0.043; 0.101]**

Heterogeneity: $Tau^2 = 0.0242$; $Chi^2 = 1172.01$, df = 29 ($P < 0.01$); $I^2 = 98\%$

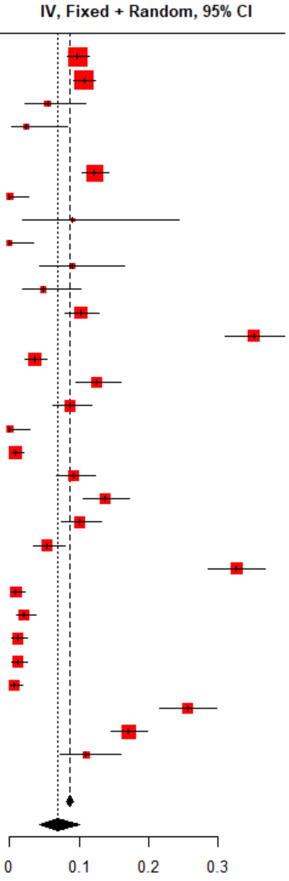

Figure S10: Forest Plot Displaying the Misdiagnosis Rate for K-L_2 Diagnosis Via DL Based on X-ray, Misclassified as K-L_0 Grade

Study	Events	Total	Weight (common)	Weight (random)	IV, Fixed + Random, 95% CI
Yassine Nasser 2023	475	1545	9.9%	3.3%	0.307 [0.284; 0.331]
Yassine Nasser 2023	365	1545	9.9%	3.3%	0.236 [0.215; 0.258]
Nan Chen 2024	15	128	0.8%	3.2%	0.117 [0.067; 0.186]
Rabbia Mahum 2021	0	85	0.5%	3.1%	0.000 [0.000; 0.042]
Berk Norman 2018	302	1306	8.4%	3.3%	0.231 [0.209; 0.255]
Kevin A. Thomas 2019	156	1114	7.1%	3.3%	0.140 [0.120; 0.162]
Jianfeng Yang 2022	1	137	0.9%	3.2%	0.007 [0.000; 0.040]
Ganesh Kumar M 2023	11	33	0.2%	2.8%	0.333 [0.180; 0.518]
S. Sheik Abdullah 2022	1	108	0.7%	3.1%	0.009 [0.000; 0.051]
Dong Hyun Kim 2020	49	99	0.6%	3.1%	0.495 [0.393; 0.597]
Dong Hyun Kim 2020	71	125	0.8%	3.2%	0.568 [0.476; 0.656]
Saleh Hamad Sajaan Almansour 2023	169	609	3.9%	3.3%	0.278 [0.242; 0.315]
Saleh Hamad Sajaan Almansour 2023	3	490	3.1%	3.3%	0.006 [0.001; 0.018]
Saleh Hamad Sajaan Almansour 2023	16	611	3.9%	3.3%	0.026 [0.015; 0.042]
Sang-min Lee 2023	58	447	2.9%	3.3%	0.130 [0.100; 0.164]
Sang-min Lee 2023	72	447	2.9%	3.3%	0.161 [0.128; 0.198]
Wei Li 2023	4	126	0.8%	3.2%	0.032 [0.009; 0.079]
Albert Swiecicki 2021	130	588	3.8%	3.3%	0.221 [0.188; 0.257]
Pingjun Chen 2019	32	447	2.9%	3.3%	0.072 [0.049; 0.100]
Pingjun Chen 2019	67	447	2.9%	3.3%	0.150 [0.118; 0.186]
Pingjun Chen 2019	85	447	2.9%	3.3%	0.190 [0.155; 0.230]
Pingjun Chen 2019	72	447	2.9%	3.3%	0.161 [0.128; 0.198]
Sozan Mohammed Ahmed 2022	43	516	3.3%	3.3%	0.083 [0.061; 0.111]
Tayyaba Tariq 2023	12	447	2.9%	3.3%	0.027 [0.014; 0.046]
Tayyaba Tariq 2023	6	447	2.9%	3.3%	0.013 [0.005; 0.029]
Tayyaba Tariq 2023	18	447	2.9%	3.3%	0.040 [0.024; 0.063]
Tayyaba Tariq 2023	11	447	2.9%	3.3%	0.025 [0.012; 0.044]
Tayyaba Tariq 2023	7	447	2.9%	3.3%	0.016 [0.006; 0.032]
Abdul Sami Mohammed 2023	23	447	2.9%	3.3%	0.051 [0.033; 0.076]
Usman Yunus 2022	3	850	5.4%	3.3%	0.004 [0.001; 0.010]
Bin Liu 2020	0	218	1.4%	3.2%	0.000 [0.000; 0.017]
**Total (common effect, 95% CI)**		**15597**	**100.0%**	--	**0.120 [0.115; 0.125]**
**Total (random effect, 95% CI)**			--	**100.0%**	**0.099 [0.059; 0.149]**

Heterogeneity: $Tau^2 = 0.0447$; $Chi^2 = 2097.89$, df = 30 (P = 0); $I^2 = 99\%$

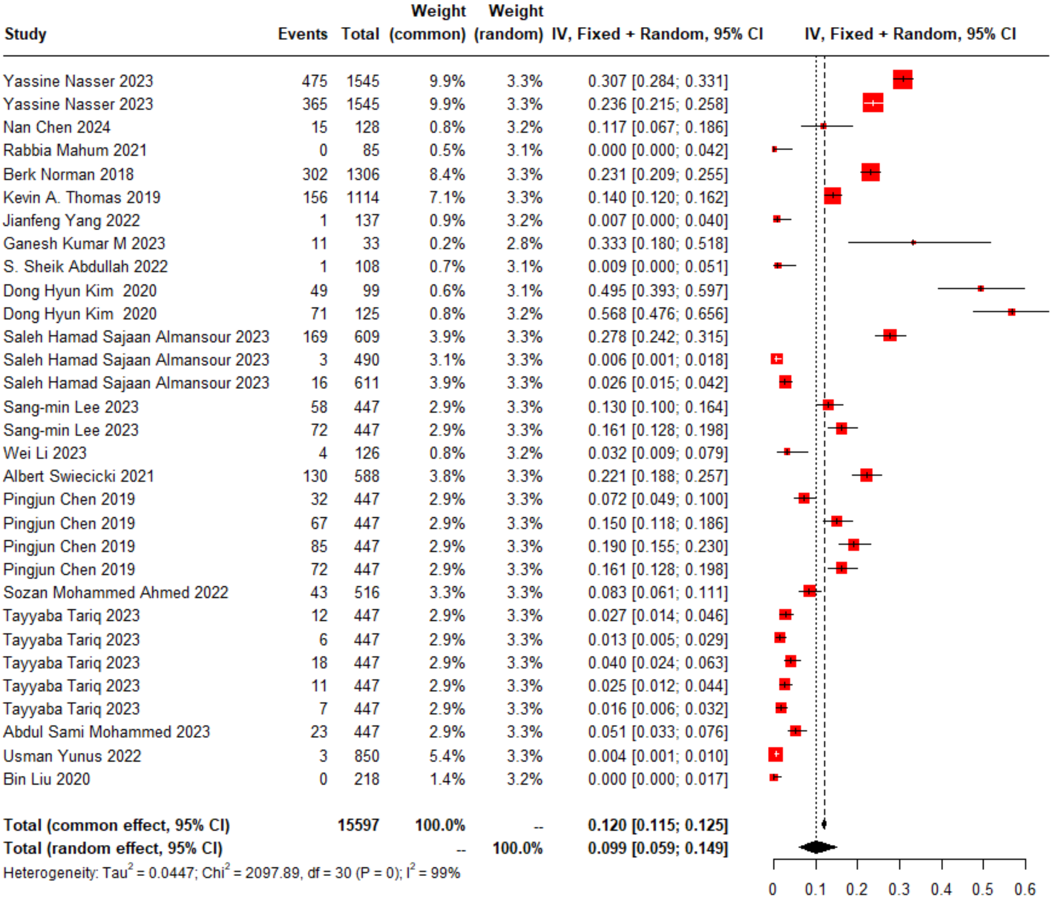

Figure S11: Forest Plot Displaying the Misdiagnosis Rate for K-L₂ Diagnosis Via DL Based on X-ray, Misclassified as K-L₁ Grade

Study	Events	Total	Weight (common)	Weight (random)	IV, Fixed + Random, 95% CI
Yassine Nasser 2023	.	1545	0.0%	0.0%	
Yassine Nasser 2023	.	1545	0.0%	0.0%	
Nan Chen 2024	12	128	1.0%	3.3%	0.094 [0.049; 0.158]
Rabbia Mahum 2021	0	85	0.7%	3.2%	0.000 [0.000; 0.042]
Berk Norman 2018	83	1306	10.4%	3.6%	0.064 [0.051; 0.078]
Kevin A. Thomas 2019	78	1114	8.9%	3.6%	0.070 [0.056; 0.087]
Jianfeng Yang 2022	2	137	1.1%	3.3%	0.015 [0.002; 0.052]
Ganesh Kumar M 2023	4	33	0.3%	2.7%	0.121 [0.034; 0.282]
S. Sheik Abdullah 2022	0	108	0.9%	3.3%	0.000 [0.000; 0.034]
Dong Hyun Kim 2020	21	99	0.8%	3.2%	0.212 [0.136; 0.306]
Dong Hyun Kim 2020	30	125	1.0%	3.3%	0.240 [0.168; 0.325]
Saleh Hamad Sajaan Almansour 2023	215	609	4.9%	3.6%	0.353 [0.315; 0.392]
Saleh Hamad Sajaan Almansour 2023	1	490	3.9%	3.5%	0.002 [0.000; 0.011]
Saleh Hamad Sajaan Almansour 2023	0	611	4.9%	3.6%	0.000 [0.000; 0.006]
Sang-min Lee 2023	17	447	3.6%	3.5%	0.038 [0.022; 0.060]
Sang-min Lee 2023	17	447	3.6%	3.5%	0.038 [0.022; 0.060]
Wei Li 2023	3	126	1.0%	3.3%	0.024 [0.005; 0.068]
Albert Swiecicki 2021	47	588	4.7%	3.6%	0.080 [0.059; 0.105]
Pingjun Chen 2019	37	447	3.6%	3.5%	0.083 [0.059; 0.112]
Pingjun Chen 2019	37	447	3.6%	3.5%	0.083 [0.059; 0.112]
Pingjun Chen 2019	25	447	3.6%	3.5%	0.056 [0.037; 0.081]
Pingjun Chen 2019	38	447	3.6%	3.5%	0.085 [0.061; 0.115]
Sozan Mohammed Ahmed 2022	31	516	4.1%	3.5%	0.060 [0.041; 0.084]
Tayyaba Tariq 2023	2	447	3.6%	3.5%	0.004 [0.001; 0.016]
Tayyaba Tariq 2023	3	447	3.6%	3.5%	0.007 [0.001; 0.019]
Tayyaba Tariq 2023	5	447	3.6%	3.5%	0.011 [0.004; 0.026]
Tayyaba Tariq 2023	6	447	3.6%	3.5%	0.013 [0.005; 0.029]
Tayyaba Tariq 2023	2	447	3.6%	3.5%	0.004 [0.001; 0.016]
Abdul Sami Mohammed 2023	33	447	3.6%	3.5%	0.074 [0.051; 0.102]
Usman Yunus 2022	1	850	6.8%	3.6%	0.001 [0.000; 0.007]
Bin Liu 2020	14	218	1.7%	3.4%	0.064 [0.036; 0.105]
**Total (common effect, 95% CI)**		**15597**	**100.0%**	--	**0.043 [0.040; 0.047]**
**Total (random effect, 95% CI)**		--		**100.0%**	**0.046 [0.025; 0.072]**

Heterogeneity: $Tau^2 = 0.0215$; $Chi^2 = 1002.90$, df = 28 ($P < 0.01$); $I^2 = 97\%$

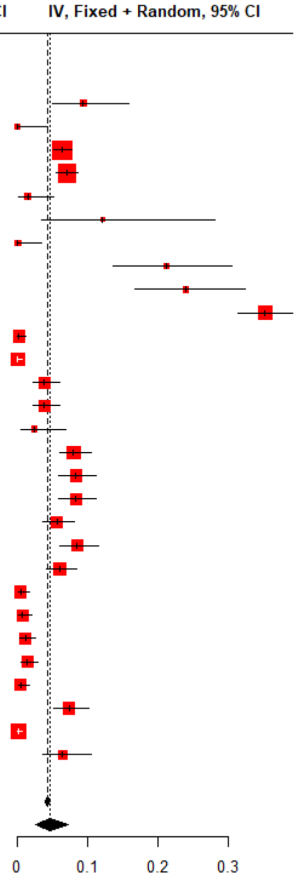

Figure S12: Forest Plot Displaying the Misdiagnosis Rate for K-L_2 Diagnosis Via DL Based on X-ray, Misclassified as K-L_3 Grade

Study	Events	Total	Weight (common)	Weight (random)	IV, Fixed + Random, 95% CI
Yassine Nasser 2023	.	1545	0.0%	0.0%	
Yassine Nasser 2023	.	1545	0.0%	0.0%	
Nan Chen 2024	0	128	1.0%	1.1%	0.000 [0.000; 0.028]
Rabbia Mahum 2021	0	85	0.7%	0.8%	0.000 [0.000; 0.042]
Berk Norman 2018	4	1306	10.4%	9.2%	0.003 [0.001; 0.008]
Kevin A. Thomas 2019	0	1114	8.9%	8.1%	0.000 [0.000; 0.003]
Jianfeng Yang 2022	0	137	1.1%	1.2%	0.000 [0.000; 0.027]
Ganesh Kumar M 2023	0	33	0.3%	0.3%	0.000 [0.000; 0.106]
S. Sheik Abdullah 2022	0	108	0.9%	1.0%	0.000 [0.000; 0.034]
Dong Hyun Kim 2020	2	99	0.8%	0.9%	0.020 [0.002; 0.071]
Dong Hyun Kim 2020	0	125	1.0%	1.1%	0.000 [0.000; 0.029]
Saleh Hamad Sajaan Almansour 2023	0	609	4.9%	4.9%	0.000 [0.000; 0.006]
Saleh Hamad Sajaan Almansour 2023	0	490	3.9%	4.0%	0.000 [0.000; 0.008]
Saleh Hamad Sajaan Almansour 2023	0	611	4.9%	4.9%	0.000 [0.000; 0.006]
Sang-min Lee 2023	0	447	3.6%	3.7%	0.000 [0.000; 0.008]
Sang-min Lee 2023	0	447	3.6%	3.7%	0.000 [0.000; 0.008]
Wei Li 2023	0	126	1.0%	1.1%	0.000 [0.000; 0.029]
Albert Swiecicki 2021	1	588	4.7%	4.7%	0.002 [0.000; 0.009]
Pingjun Chen 2019	2	447	3.6%	3.7%	0.004 [0.001; 0.016]
Pingjun Chen 2019	3	447	3.6%	3.7%	0.007 [0.001; 0.019]
Pingjun Chen 2019	0	447	3.6%	3.7%	0.000 [0.000; 0.008]
Pingjun Chen 2019	1	447	3.6%	3.7%	0.002 [0.000; 0.012]
Sozan Mohammed Ahmed 2022	1	516	4.1%	4.2%	0.002 [0.000; 0.011]
Tayyaba Tariq 2023	0	447	3.6%	3.7%	0.000 [0.000; 0.008]
Tayyaba Tariq 2023	1	447	3.6%	3.7%	0.002 [0.000; 0.012]
Tayyaba Tariq 2023	0	447	3.6%	3.7%	0.000 [0.000; 0.008]
Tayyaba Tariq 2023	0	447	3.6%	3.7%	0.000 [0.000; 0.008]
Tayyaba Tariq 2023	0	447	3.6%	3.7%	0.000 [0.000; 0.008]
Abdul Sami Mohammed 2023	1	447	3.6%	3.7%	0.002 [0.000; 0.012]
Usman Yunus 2022	0	850	6.8%	6.5%	0.000 [0.000; 0.004]
Bin Liu 2020	0	218	1.7%	1.9%	0.000 [0.000; 0.017]
**Total (common effect, 95% CI)**		**15597**	**100.0%**	--	**0.000 [0.000; 0.001]**
**Total (random effect, 95% CI)**			--	**100.0%**	**0.000 [0.000; 0.001]**

Heterogeneity: $Tau^2 < 0.0001$; $Chi^2 = 26.55$, df = 28 (P = 0.54); $I^2 = 0\%$

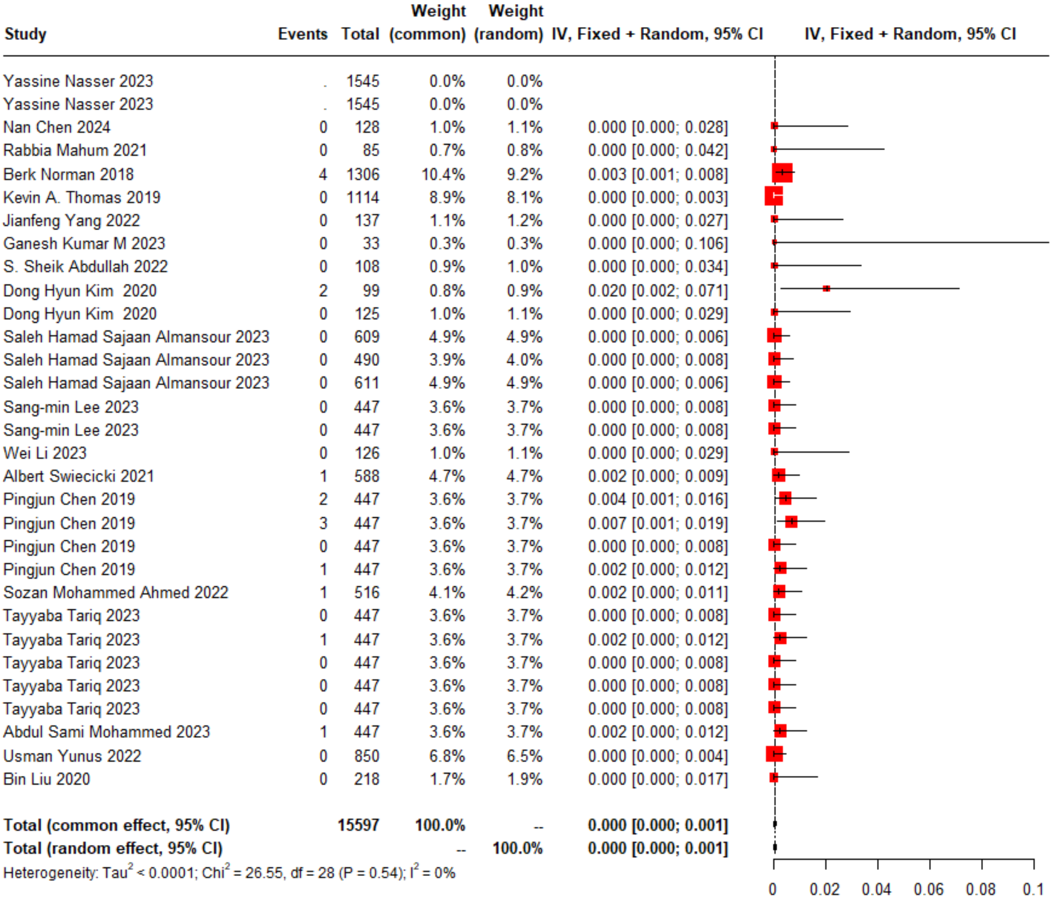

Figure S13: Forest Plot Displaying the Misdiagnosis Rate for K-L_2 Diagnosis Via DL Based on X-ray, Misclassified as K-L_4 Grade.

Study	Events	Total	Weight (common)	Weight (random)	IV, Fixed + Random, 95% CI
Nan Chen 2025	0	78	1.1%	3.0%	0.000 [0.000; 0.046]
Rabbia Mahum 2021	0	98	1.4%	3.2%	0.000 [0.000; 0.037]
Berk Norman 2018	.	791	0.0%	0.0%	
Kevin A. Thomas 2019	0	660	9.4%	4.1%	0.000 [0.000; 0.006]
Jianfeng Yang 2022	0	363	5.2%	3.9%	0.000 [0.000; 0.010]
Ganesh Kumar M 2023	1	41	0.6%	2.3%	0.024 [0.001; 0.129]
S. Sheik Abdullah 2022	0	93	1.3%	3.1%	0.000 [0.000; 0.039]
Dong Hyun Kim 2020	1	117	1.7%	3.3%	0.009 [0.000; 0.047]
Dong Hyun Kim 2020	1	147	2.1%	3.5%	0.007 [0.000; 0.037]
Saleh Hamad Sajaan Almansour 2023	0	478	6.8%	4.0%	0.000 [0.000; 0.008]
Saleh Hamad Sajaan Almansour 2023	0	490	7.0%	4.0%	0.000 [0.000; 0.008]
Saleh Hamad Sajaan Almansour 2023	0	337	4.8%	3.9%	0.000 [0.000; 0.011]
Sang-min Lee 2023	0	223	3.2%	3.7%	0.000 [0.000; 0.016]
Sang-min Lee 2023	0	223	3.2%	3.7%	0.000 [0.000; 0.016]
Wei Li 2023	0	72	1.0%	2.9%	0.000 [0.000; 0.050]
Albert Swiecicki 2021	0	697	10.0%	4.1%	0.000 [0.000; 0.005]
Pingjun Chen 2019	1	123	1.8%	3.4%	0.008 [0.000; 0.044]
Pingjun Chen 2019	3	223	3.2%	3.7%	0.013 [0.003; 0.039]
Pingjun Chen 2019	1	223	3.2%	3.7%	0.004 [0.000; 0.025]
Pingjun Chen 2019	0	223	3.2%	3.7%	0.000 [0.000; 0.016]
Sozan Mohammed Ahmed 2022	13	257	3.7%	3.8%	0.051 [0.027; 0.085]
Tayyaba Tariq 2023	0	223	3.2%	3.7%	0.000 [0.000; 0.016]
Tayyaba Tariq 2023	0	223	3.2%	3.7%	0.000 [0.000; 0.016]
Tayyaba Tariq 2023	0	223	3.2%	3.7%	0.000 [0.000; 0.016]
Tayyaba Tariq 2023	0	223	3.2%	3.7%	0.000 [0.000; 0.016]
Tayyaba Tariq 2023	0	223	3.2%	3.7%	0.000 [0.000; 0.016]
Abdul Sami Mohammed 2023	16	223	3.2%	3.7%	0.072 [0.042; 0.114]
Usman Yunus 2022	59	423	6.1%	4.0%	0.139 [0.108; 0.176]
Bin Liu 2020	0	50	0.7%	2.6%	0.000 [0.000; 0.071]
**Total (common effect, 95% CI)**		**7768**	**100.0%**	--	**0.003 [0.002; 0.005]**
**Total (random effect, 95% CI)**			--	**100.0%**	**0.004 [0.000; 0.010]**

Heterogeneity: $Tau^2 = 0.0073$; $Chi^2 = 275.35$, df = 27 ($P < 0.01$); $I^2 = 90\%$

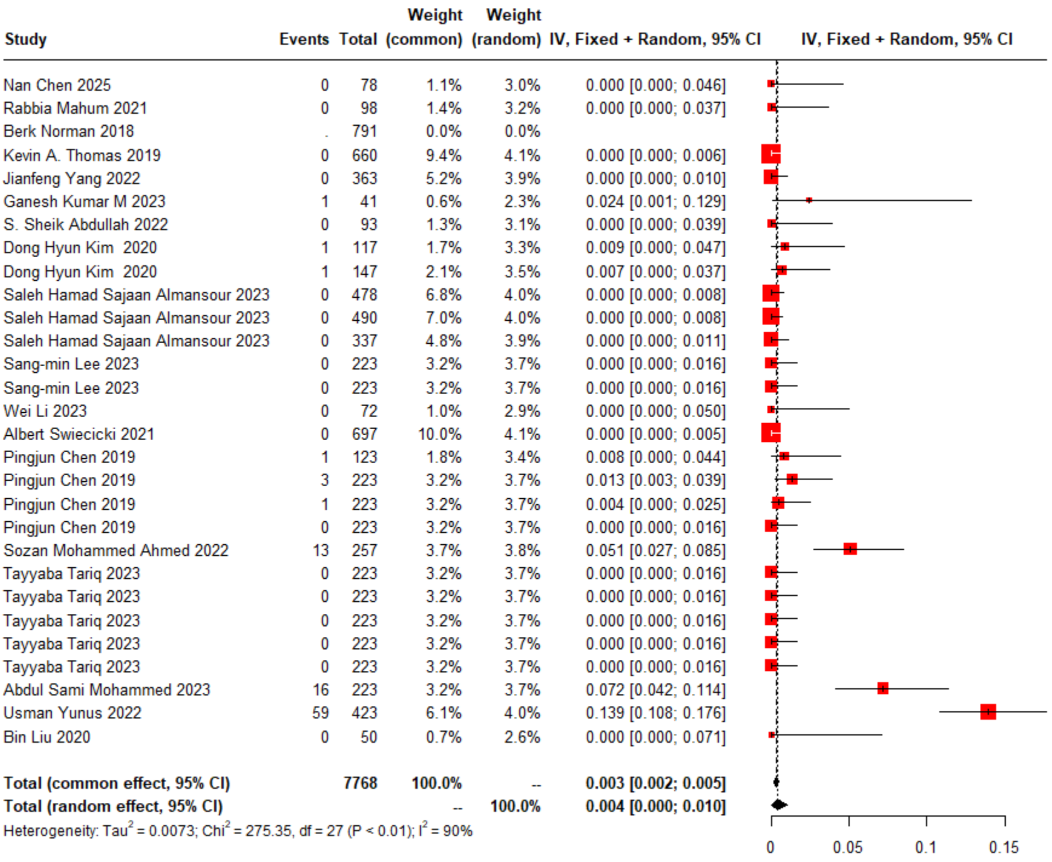

Figure S14: Forest Plot Illustrating the Misclassification Rate of K-L_3 Diagnosis Using DL Based on X-ray, Categorized as K-L_0 Grade

Study	Events	Total	Weight (common)	Weight (random)	IV, Fixed + Random, 95% CI
Nan Chen 2025	0	78	1.0%	3.1%	0.000 [0.000; 0.046]
Rabbia Mahum 2021	0	98	1.3%	3.2%	0.000 [0.000; 0.037]
Berk Norman 2018	23	791	10.2%	3.7%	0.029 [0.019; 0.043]
Kevin A. Thomas 2019	8	660	8.5%	3.7%	0.012 [0.005; 0.024]
Jianfeng Yang 2022	0	363	4.7%	3.6%	0.000 [0.000; 0.010]
Ganesh Kumar M 2023	4	41	0.5%	2.7%	0.098 [0.027; 0.231]
S. Sheik Abdullah 2022	0	93	1.2%	3.2%	0.000 [0.000; 0.039]
Dong Hyun Kim 2020	18	117	1.5%	3.3%	0.154 [0.094; 0.232]
Dong Hyun Kim 2020	30	147	1.9%	3.4%	0.204 [0.142; 0.278]
Saleh Hamad Sajaan Almansour 2023	0	478	6.1%	3.7%	0.000 [0.000; 0.008]
Saleh Hamad Sajaan Almansour 2023	78	490	6.3%	3.7%	0.159 [0.128; 0.195]
Saleh Hamad Sajaan Almansour 2023	0	337	4.3%	3.6%	0.000 [0.000; 0.011]
Sang-min Lee 2023	10	223	2.9%	3.5%	0.045 [0.022; 0.081]
Sang-min Lee 2023	7	223	2.9%	3.5%	0.031 [0.013; 0.064]
Wei Li 2023	0	72	0.9%	3.1%	0.000 [0.000; 0.050]
Albert Swiecicki 2021	11	697	9.0%	3.7%	0.016 [0.008; 0.028]
Pingjun Chen 2019	1	123	1.6%	3.3%	0.008 [0.000; 0.044]
Pingjun Chen 2019	4	223	2.9%	3.5%	0.018 [0.005; 0.045]
Pingjun Chen 2019	6	223	2.9%	3.5%	0.027 [0.010; 0.058]
Pingjun Chen 2019	5	223	2.9%	3.5%	0.022 [0.007; 0.052]
Sozan Mohammed Ahmed 2022	7	257	3.3%	3.6%	0.027 [0.011; 0.055]
Tayyaba Tariq 2023	0	223	2.9%	3.5%	0.000 [0.000; 0.016]
Tayyaba Tariq 2023	0	223	2.9%	3.5%	0.000 [0.000; 0.016]
Tayyaba Tariq 2023	0	223	2.9%	3.5%	0.000 [0.000; 0.016]
Tayyaba Tariq 2023	0	223	2.9%	3.5%	0.000 [0.000; 0.016]
Tayyaba Tariq 2023	0	223	2.9%	3.5%	0.000 [0.000; 0.016]
Abdul Sami Mohammed 2023	5	223	2.9%	3.5%	0.022 [0.007; 0.052]
Usman Yunus 2022	4	423	5.4%	3.7%	0.009 [0.003; 0.024]
Bin Liu 2020	0	50	0.6%	2.8%	0.000 [0.000; 0.071]
**Total (common effect, 95% CI)**		**7768**	**100.0%**	--	**0.015 [0.012; 0.018]**
**Total (random effect, 95% CI)**			--	**100.0%**	**0.016 [0.006; 0.030]**

Heterogeneity: $Tau^2 = 0.0137$; $Chi^2 = 390.54$, df = 28 ($P < 0.01$); $I^2 = 93\%$

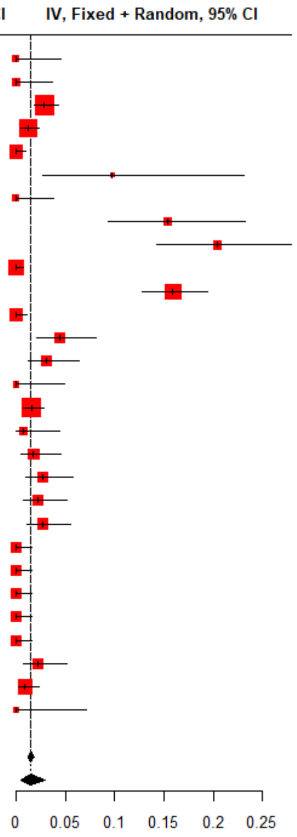

Figure S15: Forest Plot Illustrating the Misclassification Rate of K-L_3 Diagnosis Using DL Based on X-ray, Categorized as K-L_1 Grade

Study	Events	Total	Weight (common)	Weight (random)	IV, Fixed + Random, 95% CI
Nan Chen 2025	7	78	1.0%	3.3%	0.090 [0.037; 0.176]
Rabbia Mahum 2021	0	98	1.3%	3.3%	0.000 [0.000; 0.037]
Berk Norman 2018	183	791	10.2%	3.6%	0.231 [0.202; 0.262]
Kevin A. Thomas 2019	91	660	8.5%	3.6%	0.138 [0.112; 0.167]
Jianfeng Yang 2022	1	363	4.7%	3.5%	0.003 [0.000; 0.015]
Ganesh Kumar M 2023	2	41	0.5%	3.0%	0.049 [0.006; 0.165]
S. Sheik Abdullah 2022	0	93	1.2%	3.3%	0.000 [0.000; 0.039]
Dong Hyun Kim 2020	14	117	1.5%	3.4%	0.120 [0.067; 0.193]
Dong Hyun Kim 2020	9	147	1.9%	3.4%	0.061 [0.028; 0.113]
Saleh Hamad Sajaan Almansour 2023	52	478	6.1%	3.6%	0.109 [0.082; 0.140]
Saleh Hamad Sajaan Almansour 2023	0	490	6.3%	3.6%	0.000 [0.000; 0.008]
Saleh Hamad Sajaan Almansour 2023	3	337	4.3%	3.5%	0.009 [0.002; 0.026]
Sang-min Lee 2023	25	223	2.9%	3.5%	0.112 [0.074; 0.161]
Sang-min Lee 2023	34	223	2.9%	3.5%	0.152 [0.108; 0.206]
Wei Li 2023	4	72	0.9%	3.3%	0.056 [0.015; 0.136]
Albert Swiecicki 2021	102	697	9.0%	3.6%	0.146 [0.121; 0.175]
Pingjun Chen 2019	33	123	1.6%	3.4%	0.268 [0.192; 0.356]
Pingjun Chen 2019	26	223	2.9%	3.5%	0.117 [0.078; 0.166]
Pingjun Chen 2019	37	223	2.9%	3.5%	0.166 [0.120; 0.221]
Pingjun Chen 2019	27	223	2.9%	3.5%	0.121 [0.081; 0.171]
Sozan Mohammed Ahmed 2022	46	257	3.3%	3.5%	0.179 [0.134; 0.231]
Tayyaba Tariq 2023	7	223	2.9%	3.5%	0.031 [0.013; 0.064]
Tayyaba Tariq 2023	3	223	2.9%	3.5%	0.013 [0.003; 0.039]
Tayyaba Tariq 2023	6	223	2.9%	3.5%	0.027 [0.010; 0.058]
Tayyaba Tariq 2023	3	223	2.9%	3.5%	0.013 [0.003; 0.039]
Tayyaba Tariq 2023	5	223	2.9%	3.5%	0.022 [0.007; 0.052]
Abdul Sami Mohammed 2023	37	223	2.9%	3.5%	0.166 [0.120; 0.221]
Usman Yunus 2022	0	423	5.4%	3.5%	0.000 [0.000; 0.009]
Bin Liu 2020	28	50	0.6%	3.1%	0.560 [0.413; 0.700]
**Total (common effect, 95% CI)**		**7768**	**100.0%**	--	**0.076 [0.070; 0.082]**
**Total (random effect, 95% CI)**			--	**100.0%**	**0.075 [0.043; 0.114]**

Heterogeneity: $Tau^2 = 0.0316$; $Chi^2 = 884.76$, df = 28 ($P < 0.01$); $I^2 = 97\%$

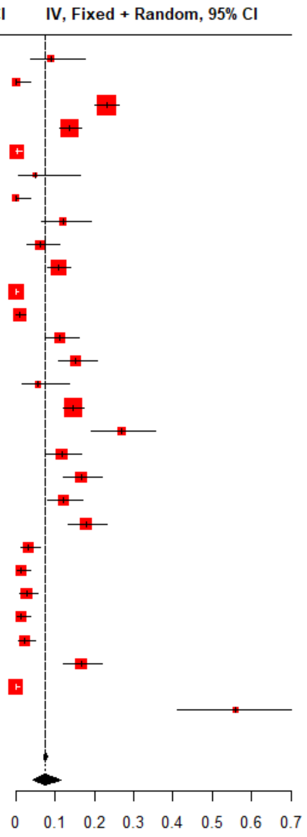

Figure S16: Forest Plot Illustrating the Misclassification Rate of K-L3 Diagnosis Using DL Based on X-ray, Categorized as K-L2 Grade.

Study	Events	Total	Weight (common)	Weight (random)	IV, Fixed + Random, 95% CI
Nan Chen 2025	4	78	1.0%	3.1%	0.051 [0.014; 0.126]
Rabbia Mahum 2021	0	98	1.3%	3.2%	0.000 [0.000; 0.037]
Berk Norman 2018	40	791	10.2%	3.7%	0.051 [0.036; 0.068]
Kevin A. Thomas 2019	27	660	8.5%	3.7%	0.041 [0.027; 0.059]
Jianfeng Yang 2022	6	363	4.7%	3.6%	0.017 [0.006; 0.036]
Ganesh Kumar M 2023	3	41	0.5%	2.7%	0.073 [0.015; 0.199]
S. Sheik Abdullah 2022	0	93	1.2%	3.2%	0.000 [0.000; 0.039]
Dong Hyun Kim 2020	23	117	1.5%	3.3%	0.197 [0.129; 0.280]
Dong Hyun Kim 2020	14	147	1.9%	3.4%	0.095 [0.053; 0.155]
Saleh Hamad Sajaan Almansour 2023	62	478	6.1%	3.7%	0.130 [0.101; 0.163]
Saleh Hamad Sajaan Almansour 2023	15	490	6.3%	3.7%	0.031 [0.017; 0.050]
Saleh Hamad Sajaan Almansour 2023	0	337	4.3%	3.6%	0.000 [0.000; 0.011]
Sang-min Lee 2023	4	223	2.9%	3.5%	0.018 [0.005; 0.045]
Sang-min Lee 2023	6	223	2.9%	3.5%	0.027 [0.010; 0.058]
Wei Li 2023	1	72	0.9%	3.1%	0.014 [0.000; 0.075]
Albert Swiecicki 2021	44	697	9.0%	3.7%	0.063 [0.046; 0.084]
Pingjun Chen 2019	21	123	1.6%	3.3%	0.171 [0.109; 0.249]
Pingjun Chen 2019	18	223	2.9%	3.5%	0.081 [0.049; 0.125]
Pingjun Chen 2019	4	223	2.9%	3.5%	0.018 [0.005; 0.045]
Pingjun Chen 2019	11	223	2.9%	3.5%	0.049 [0.025; 0.087]
Sozan Mohammed Ahmed 2022	6	257	3.3%	3.6%	0.023 [0.009; 0.050]
Tayyaba Tariq 2023	0	223	2.9%	3.5%	0.000 [0.000; 0.016]
Tayyaba Tariq 2023	0	223	2.9%	3.5%	0.000 [0.000; 0.016]
Tayyaba Tariq 2023	0	223	2.9%	3.5%	0.000 [0.000; 0.016]
Tayyaba Tariq 2023	0	223	2.9%	3.5%	0.000 [0.000; 0.016]
Tayyaba Tariq 2023	0	223	2.9%	3.5%	0.000 [0.000; 0.016]
Abdul Sami Mohammed 2023	4	223	2.9%	3.5%	0.018 [0.005; 0.045]
Usman Yunus 2022	0	423	5.4%	3.7%	0.000 [0.000; 0.009]
Bin Liu 2020	4	50	0.6%	2.8%	0.080 [0.022; 0.192]
**Total (common effect, 95% CI)**		**7768**	**100.0%**	--	**0.028 [0.024; 0.032]**
**Total (random effect, 95% CI)**			--	**100.0%**	**0.028 [0.014; 0.045]**

Heterogeneity: $Tau^2 = 0.0142$; $Chi^2 = 381.55$, df = 28 ($P < 0.01$); $I^2 = 93\%$

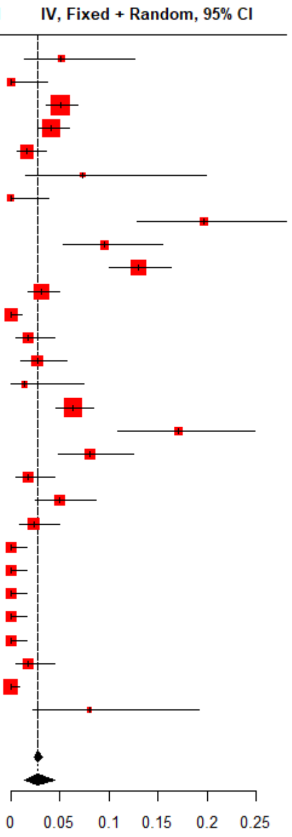

Figure S17: Forest Plot Illustrating the Misclassification Rate of K-L3 Diagnosis Using DL Based on X-ray, Categorized as K-L4 Grade.

Study	Events	Total	Weight (common)	Weight (random)	IV, Fixed + Random, 95% CI
Nan Chen 2026	0	81	3.0%	4.0%	0.000 [0.000; 0.045]
Rabbia Mahum 2021	0	104	3.9%	4.4%	0.000 [0.000; 0.035]
Berk Norman 2018	.	235	0.0%	0.0%	
Kevin A. Thomas 2019	0	219	8.2%	5.5%	0.000 [0.000; 0.017]
Jianfeng Yang 2022	0	339	12.7%	6.0%	0.000 [0.000; 0.011]
Ganesh Kumar M 2023	0	9	0.4%	0.9%	0.000 [0.000; 0.336]
S. Sheik Abdullah 2022	0	91	3.4%	4.2%	0.000 [0.000; 0.040]
Dong Hyun Kim 2020	0	53	2.0%	3.2%	0.000 [0.000; 0.067]
Dong Hyun Kim 2020	0	53	2.0%	3.2%	0.000 [0.000; 0.067]
Saleh Hamad Sajaan Almansour 2023	0	96	3.6%	4.3%	0.000 [0.000; 0.038]
Saleh Hamad Sajaan Almansour 2023	0	493	18.4%	6.4%	0.000 [0.000; 0.007]
Saleh Hamad Sajaan Almansour 2023	0	71	2.7%	3.7%	0.000 [0.000; 0.051]
Sang-min Lee 2023	0	51	1.9%	3.2%	0.000 [0.000; 0.070]
Sang-min Lee 2023	0	51	1.9%	3.2%	0.000 [0.000; 0.070]
Wei Li 2023	0	31	1.2%	2.3%	0.000 [0.000; 0.112]
Albert Swiecicki 2021	0	265	9.9%	5.8%	0.000 [0.000; 0.014]
Pingjun Chen 2019	0	51	1.9%	3.2%	0.000 [0.000; 0.070]
Pingjun Chen 2019	0	10	0.4%	1.0%	0.000 [0.000; 0.308]
Pingjun Chen 2019	0	51	1.9%	3.2%	0.000 [0.000; 0.070]
Pingjun Chen 2019	0	50	1.9%	3.1%	0.000 [0.000; 0.071]
Sozan Mohammed Ahmed 2022	0	59	2.2%	3.4%	0.000 [0.000; 0.061]
Tayyaba Tariq 2023	0	51	1.9%	3.2%	0.000 [0.000; 0.070]
Tayyaba Tariq 2023	0	51	1.9%	3.2%	0.000 [0.000; 0.070]
Tayyaba Tariq 2023	0	51	1.9%	3.2%	0.000 [0.000; 0.070]
Tayyaba Tariq 2023	0	51	1.9%	3.2%	0.000 [0.000; 0.070]
Tayyaba Tariq 2023	0	51	1.9%	3.2%	0.000 [0.000; 0.070]
Abdul Sami Mohammed 2023	0	51	1.9%	3.2%	0.000 [0.000; 0.070]
Usman Yunus 2022	16	95	3.6%	4.3%	0.168 [0.099; 0.259]
Bin Liu 2020	0	36	1.4%	2.6%	0.000 [0.000; 0.097]
**Total (common effect, 95% CI)**		**2900**	**100.0%**	--	**0.000 [0.000; 0.000]**
**Total (random effect, 95% CI)**			--	**100.0%**	**0.000 [0.000; 0.003]**

Heterogeneity: Tau^2 = 0.0038; Chi^2 = 58.62, df = 27 (P < 0.01); I^2 = 54%

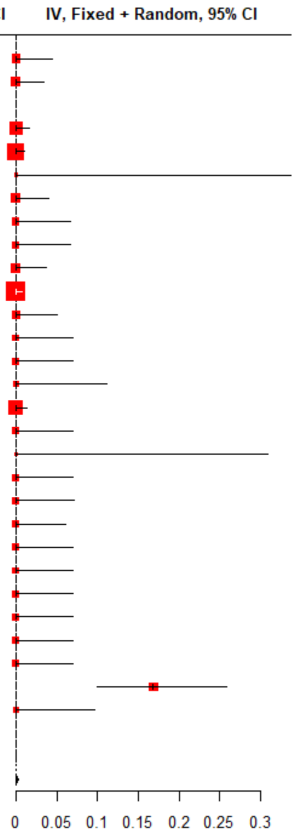

Figure S18: Forest Plot Demonstrating the Misclassification Rate for K-L$_4$ Diagnosis Using DL Based on X-ray, Representing Misidentification as K-L$_0$ Grade.

Study	Events	Total	Weight (common)	Weight (random)	IV, Fixed + Random, 95% CI
Nan Chen 2026	0	81	2.8%	2.8%	0.000 [0.000; 0.045]
Rabbia Mahum 2021	0	104	3.6%	3.6%	0.000 [0.000; 0.035]
Berk Norman 2018	0	235	8.1%	8.1%	0.000 [0.000; 0.016]
Kevin A. Thomas 2019	0	219	7.5%	7.5%	0.000 [0.000; 0.017]
Jianfeng Yang 2022	0	339	11.6%	11.6%	0.000 [0.000; 0.011]
Ganesh Kumar M 2023	0	9	0.3%	0.3%	0.000 [0.000; 0.336]
S. Sheik Abdullah 2022	0	91	3.1%	3.1%	0.000 [0.000; 0.040]
Dong Hyun Kim 2020	0	53	1.8%	1.8%	0.000 [0.000; 0.067]
Dong Hyun Kim 2020	0	53	1.8%	1.8%	0.000 [0.000; 0.067]
Saleh Hamad Sajaan Almansour 2023	0	96	3.3%	3.3%	0.000 [0.000; 0.038]
Saleh Hamad Sajaan Almansour 2023	0	493	16.9%	16.9%	0.000 [0.000; 0.007]
Saleh Hamad Sajaan Almansour 2023	0	71	2.5%	2.5%	0.000 [0.000; 0.051]
Sang-min Lee 2023	0	51	1.8%	1.8%	0.000 [0.000; 0.070]
Sang-min Lee 2023	0	51	1.8%	1.8%	0.000 [0.000; 0.070]
Wei Li 2023	0	31	1.1%	1.1%	0.000 [0.000; 0.112]
Albert Swiecicki 2021	0	265	9.1%	9.1%	0.000 [0.000; 0.014]
Pingjun Chen 2019	0	51	1.8%	1.8%	0.000 [0.000; 0.070]
Pingjun Chen 2019	0	10	0.4%	0.4%	0.000 [0.000; 0.308]
Pingjun Chen 2019	0	51	1.8%	1.8%	0.000 [0.000; 0.070]
Pingjun Chen 2019	0	50	1.7%	1.7%	0.000 [0.000; 0.071]
Sozan Mohammed Ahmed 2022	0	59	2.0%	2.0%	0.000 [0.000; 0.061]
Tayyaba Tariq 2023	0	51	1.8%	1.8%	0.000 [0.000; 0.070]
Tayyaba Tariq 2023	0	51	1.8%	1.8%	0.000 [0.000; 0.070]
Tayyaba Tariq 2023	0	51	1.8%	1.8%	0.000 [0.000; 0.070]
Tayyaba Tariq 2023	0	51	1.8%	1.8%	0.000 [0.000; 0.070]
Tayyaba Tariq 2023	0	51	1.8%	1.8%	0.000 [0.000; 0.070]
Abdul Sami Mohammed 2023	0	51	1.8%	1.8%	0.000 [0.000; 0.070]
Usman Yunus 2022	1	95	3.3%	3.3%	0.011 [0.000; 0.057]
Bin Liu 2020	0	36	1.3%	1.3%	0.000 [0.000; 0.097]
**Total (common effect, 95% CI)**		**2900**	**100.0%**	--	**0.000 [0.000; 0.000]**
**Total (random effect, 95% CI)**			--	**100.0%**	**0.000 [0.000; 0.000]**

Heterogeneity: $Tau^2 = 0$; $Chi^2 = 7.47$, df = 28 (P = 1.00); $I^2 = 0\%$

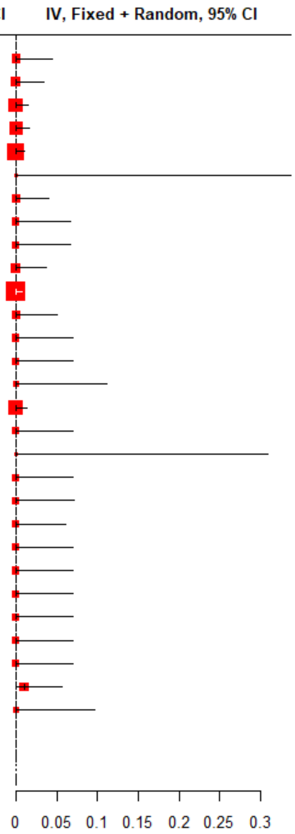

Figure S19: Forest Plot Highlighting the Misclassification Rate for K-L4 Diagnosis via DL Techniques on X-ray Imagery, Indicating Misidentification as K-L1 Grade.

Study	Events	Total	Weight (common)	Weight (random)	IV, Fixed + Random, 95% CI
Nan Chen 2026	0	81	2.8%	3.7%	0.000 [0.000; 0.045]
Rabbia Mahum 2021	0	104	3.6%	4.3%	0.000 [0.000; 0.035]
Berk Norman 2018	0	235	8.1%	6.1%	0.000 [0.000; 0.016]
Kevin A. Thomas 2019	0	219	7.5%	5.9%	0.000 [0.000; 0.017]
Jianfeng Yang 2022	0	339	11.6%	6.8%	0.000 [0.000; 0.011]
Ganesh Kumar M 2023	0	9	0.3%	0.7%	0.000 [0.000; 0.336]
S. Sheik Abdullah 2022	0	91	3.1%	3.9%	0.000 [0.000; 0.040]
Dong Hyun Kim 2020	0	53	1.8%	2.8%	0.000 [0.000; 0.067]
Dong Hyun Kim 2020	1	53	1.8%	2.8%	0.019 [0.000; 0.101]
Saleh Hamad Sajaan Almansour 2023	0	96	3.3%	4.1%	0.000 [0.000; 0.038]
Saleh Hamad Sajaan Almansour 2023	0	493	16.9%	7.4%	0.000 [0.000; 0.007]
Saleh Hamad Sajaan Almansour 2023	0	71	2.5%	3.4%	0.000 [0.000; 0.051]
Sang-min Lee 2023	0	51	1.8%	2.7%	0.000 [0.000; 0.070]
Sang-min Lee 2023	1	51	1.8%	2.7%	0.020 [0.000; 0.104]
Wei Li 2023	0	31	1.1%	1.9%	0.000 [0.000; 0.112]
Albert Swiecicki 2021	0	265	9.1%	6.3%	0.000 [0.000; 0.014]
Pingjun Chen 2019	1	51	1.8%	2.7%	0.020 [0.000; 0.104]
Pingjun Chen 2019	1	10	0.4%	0.7%	0.100 [0.003; 0.445]
Pingjun Chen 2019	1	51	1.8%	2.7%	0.020 [0.000; 0.104]
Pingjun Chen 2019	0	50	1.7%	2.7%	0.000 [0.000; 0.071]
Sozan Mohammed Ahmed 2022	5	59	2.0%	3.0%	0.085 [0.028; 0.187]
Tayyaba Tariq 2023	0	51	1.8%	2.7%	0.000 [0.000; 0.070]
Tayyaba Tariq 2023	0	51	1.8%	2.7%	0.000 [0.000; 0.070]
Tayyaba Tariq 2023	0	51	1.8%	2.7%	0.000 [0.000; 0.070]
Tayyaba Tariq 2023	0	51	1.8%	2.7%	0.000 [0.000; 0.070]
Tayyaba Tariq 2023	0	51	1.8%	2.7%	0.000 [0.000; 0.070]
Abdul Sami Mohammed 2023	4	51	1.8%	2.7%	0.078 [0.022; 0.189]
Usman Yunus 2022	1	95	3.3%	4.0%	0.011 [0.000; 0.057]
Bin Liu 2020	0	36	1.3%	2.1%	0.000 [0.000; 0.097]
**Total (common effect, 95% CI)**		**2900**	**100.0%**	--	**0.000 [0.000; 0.000]**
**Total (random effect, 95% CI)**			--	**100.0%**	**0.000 [0.000; 0.004]**

Heterogeneity: $Tau^2 = 0.0021$; $Chi^2 = 48.60$, df = 28 ($P < 0.01$); $I^2 = 42\%$

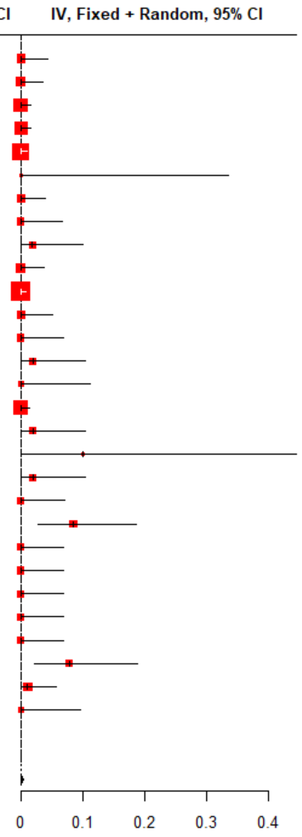

Figure S20: Forest Plot Illustrating the Misclassification Rate for K-L4 Diagnosis Using DL Based on X-ray, Portraying Misidentification as K-L2 Grade.

Study	Events	Total	Weight (common)	Weight (random)	IV, Fixed + Random, 95% CI
Nan Chen 2026	0	81	2.8%	3.6%	0.000 [0.000; 0.045]
Rabbia Mahum 2021	0	104	3.6%	3.7%	0.000 [0.000; 0.035]
Berk Norman 2018	33	235	8.1%	3.8%	0.140 [0.099; 0.192]
Kevin A. Thomas 2019	31	219	7.5%	3.8%	0.142 [0.098; 0.195]
Jianfeng Yang 2022	4	339	11.6%	3.9%	0.012 [0.003; 0.030]
Ganesh Kumar M 2023	1	9	0.3%	2.1%	0.111 [0.003; 0.482]
S. Sheik Abdullah 2022	3	91	3.1%	3.6%	0.033 [0.007; 0.093]
Dong Hyun Kim 2020	14	53	1.8%	3.4%	0.264 [0.153; 0.403]
Dong Hyun Kim 2020	17	53	1.8%	3.4%	0.321 [0.199; 0.463]
Saleh Hamad Sajaan Almansour 2023	0	96	3.3%	3.7%	0.000 [0.000; 0.038]
Saleh Hamad Sajaan Almansour 2023	22	493	16.9%	3.9%	0.045 [0.028; 0.067]
Saleh Hamad Sajaan Almansour 2023	0	71	2.5%	3.6%	0.000 [0.000; 0.051]
Sang-min Lee 2023	6	51	1.8%	3.4%	0.118 [0.044; 0.239]
Sang-min Lee 2023	8	51	1.8%	3.4%	0.157 [0.070; 0.286]
Wei Li 2023	2	31	1.1%	3.2%	0.065 [0.008; 0.214]
Albert Swiecicki 2021	18	265	9.1%	3.9%	0.068 [0.041; 0.105]
Pingjun Chen 2019	7	51	1.8%	3.4%	0.137 [0.057; 0.263]
Pingjun Chen 2019	5	10	0.4%	2.2%	0.500 [0.187; 0.813]
Pingjun Chen 2019	19	51	1.8%	3.4%	0.373 [0.241; 0.519]
Pingjun Chen 2019	8	50	1.7%	3.4%	0.160 [0.072; 0.291]
Sozan Mohammed Ahmed 2022	13	59	2.0%	3.5%	0.220 [0.123; 0.347]
Tayyaba Tariq 2023	2	51	1.8%	3.4%	0.039 [0.005; 0.135]
Tayyaba Tariq 2023	3	51	1.8%	3.4%	0.059 [0.012; 0.162]
Tayyaba Tariq 2023	2	51	1.8%	3.4%	0.039 [0.005; 0.135]
Tayyaba Tariq 2023	4	51	1.8%	3.4%	0.078 [0.022; 0.189]
Tayyaba Tariq 2023	4	51	1.8%	3.4%	0.078 [0.022; 0.189]
Abdul Sami Mohammed 2023	12	51	1.8%	3.4%	0.235 [0.128; 0.375]
Usman Yunus 2022	0	95	3.3%	3.7%	0.000 [0.000; 0.038]
Bin Liu 2020	0	36	1.3%	3.2%	0.000 [0.000; 0.097]
**Total (common effect, 95% CI)**		**2900**	**100.0%**	--	**0.057 [0.048; 0.066]**
**Total (random effect, 95% CI)**			--	**100.0%**	**0.080 [0.045; 0.124]**

Heterogeneity: $Tau^2 = 0.0306$; $Chi^2 = 276.67$, df = 28 ($P < 0.01$); $I^2 = 90\%$

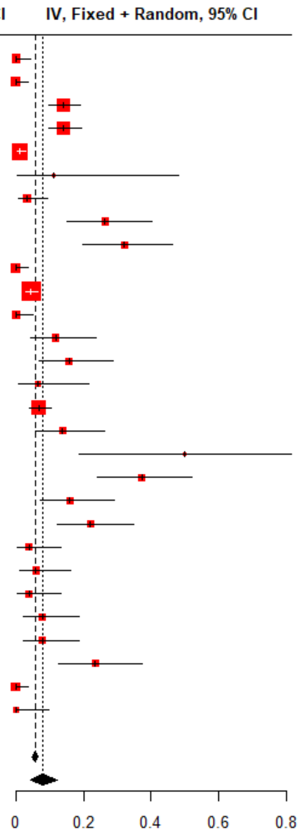

Figure S21: Forest Plot Showcasing the Misclassification Rate for K-L_4 Diagnosis Through DL on X-ray Imagery, Signifying Misidentification as K-L_3 Grade.

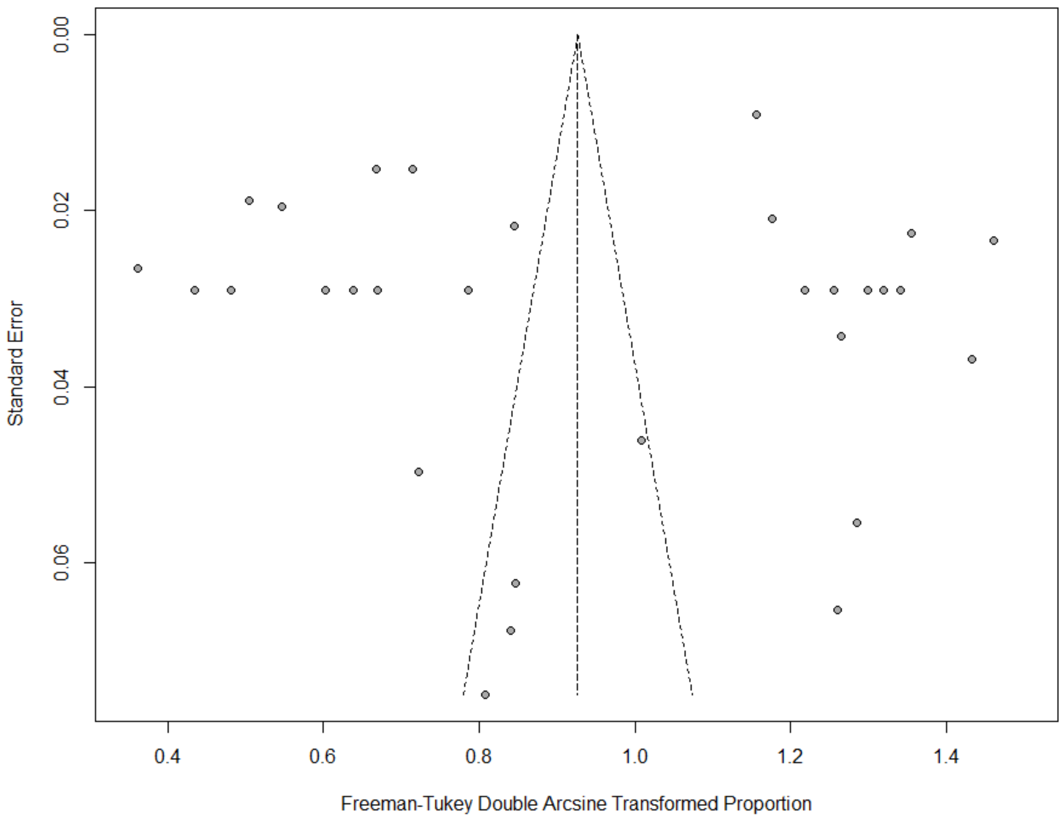

Figure S22 Funnel plot for K-L0

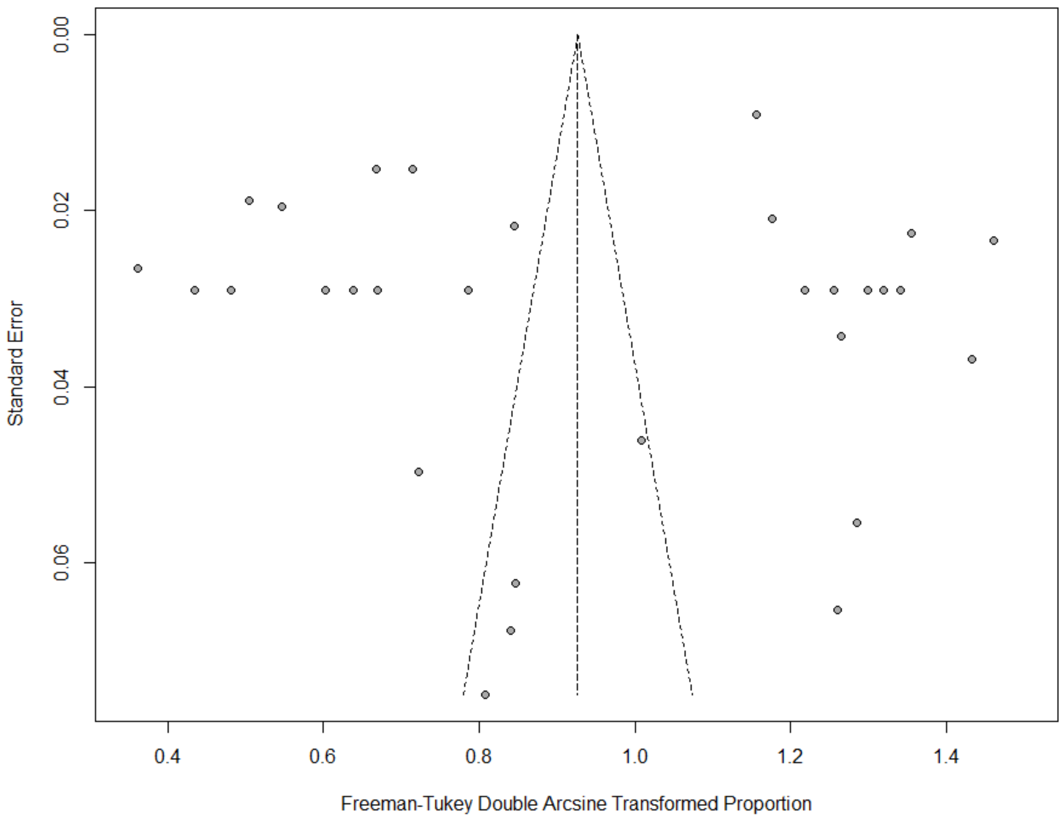

Figure S23 Funnel plot for K-L1

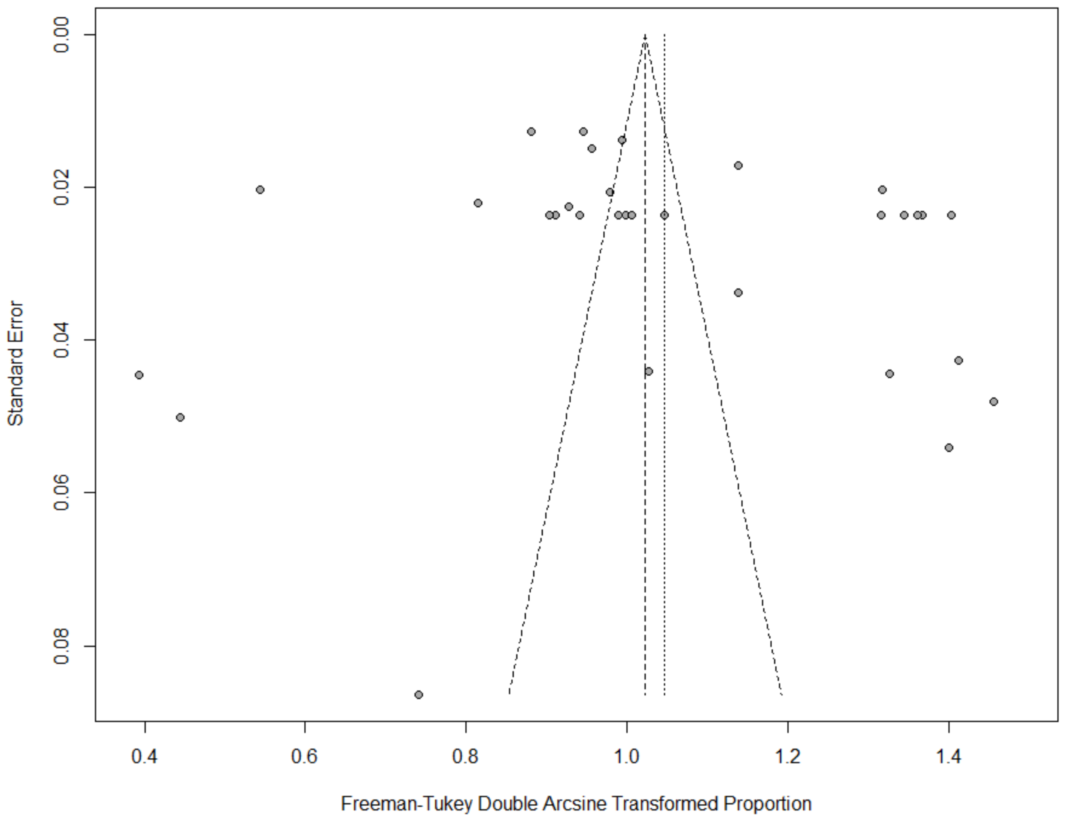

Figure S24 Funnel plot for K-L2

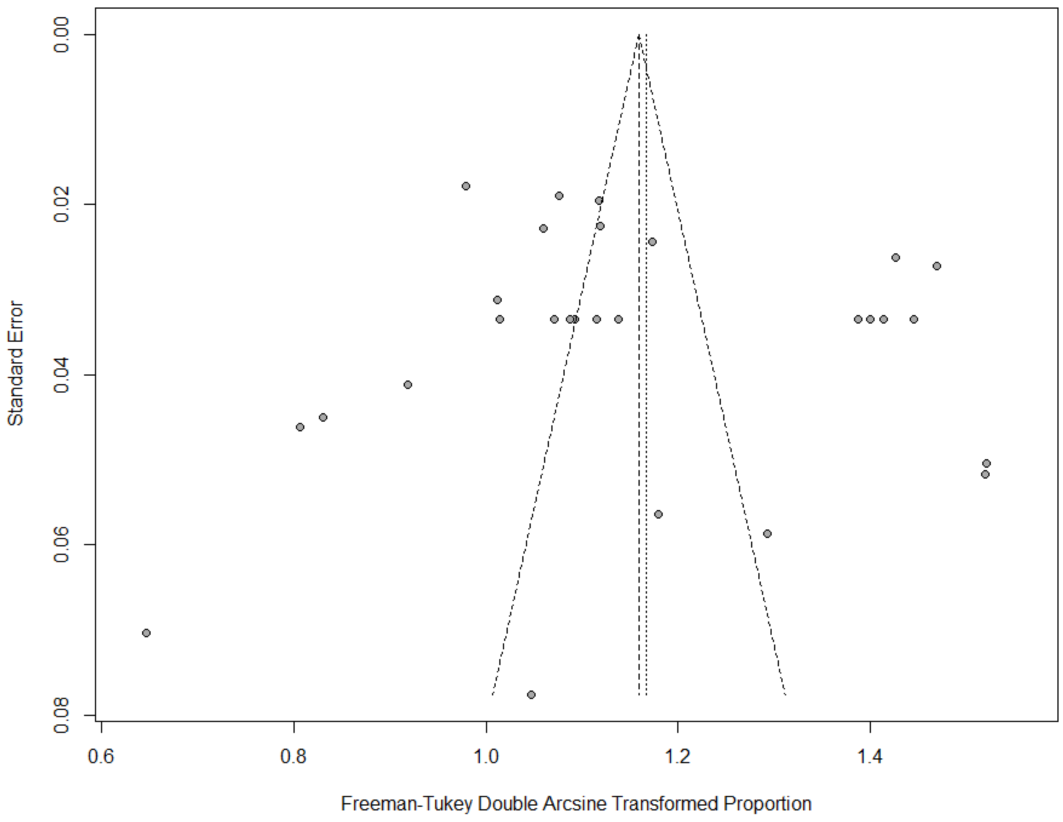

Figure S25 Funnel plot for K-L3

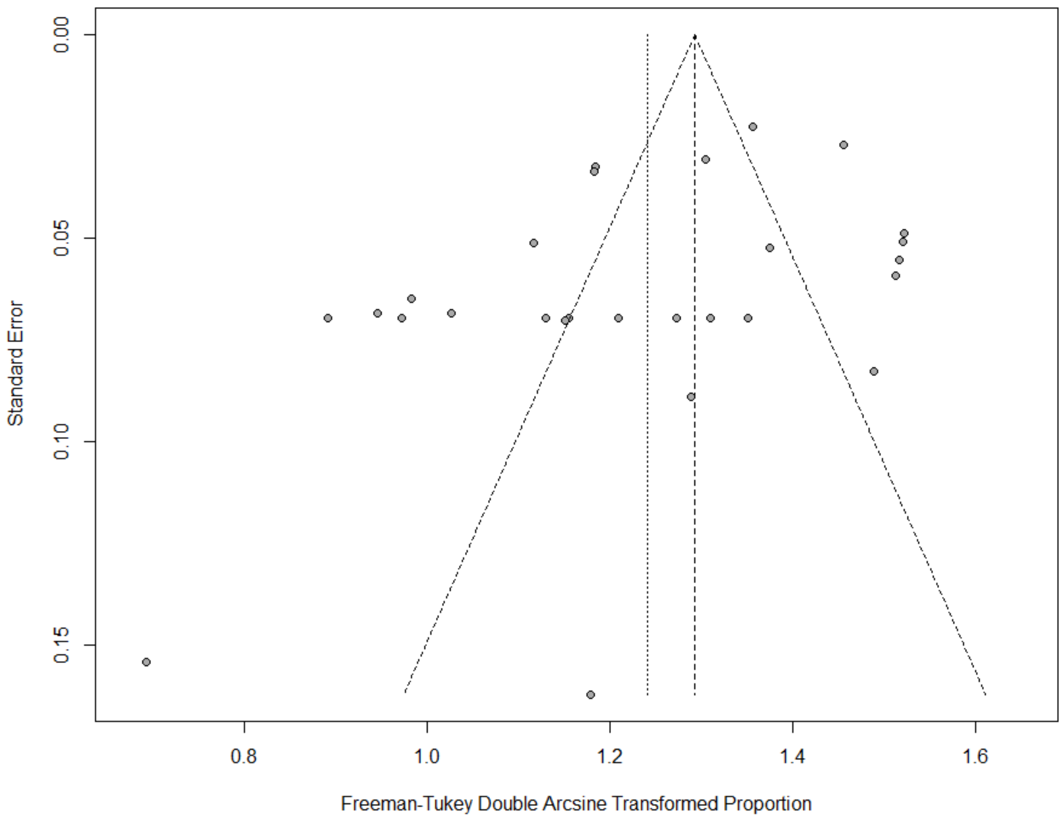

Figure S26 Funnel plot for K-L4
